# Super-enhancer-driven AJUBA is activated by TCF4 and involved in epithelial-mesenchymal transition in the progression of Hepatocellular Carcinoma

**DOI:** 10.7150/thno.45349

**Published:** 2020-07-11

**Authors:** Chi Zhang, Shi Wei, Wei-Peng Sun, Kai Teng, Miao-Miao Dai, Feng-Wei Wang, Jie-Wei Chen, Han Ling, Xiao-Dan Ma, Zi-Hao Feng, Jin-Ling Duan, Mu-Yan Cai, Dan Xie

**Affiliations:** 1 State Key Laboratory of Oncology in South China, Collaborative Innovation Center for Cancer Medicine, Sun Yat-sen University Cancer Center, 510060, Guangzhou, China.; 2 Department of Anorectal Surgery, The First Affiliated Hospital of Zhengzhou University, 450052, Zhengzhou, China.; 3 Department of Radiation Oncology, Shandong Cancer Hospital and Institute, Shandong First Medical University and Shandong Academy of Medical Sciences, 271000, Jinan, China.; 4 Department of Pathology, Sun Yat-sen University Cancer Center, 510060, Guangzhou, China.; 5 Department of Urology, First Affiliated Hospital, Sun Yat-sen University, 510060, Guangzhou, China.

**Keywords:** super-enhancer, AJUBA, TCF4, EMT, hepatocellular carcinoma

## Abstract

**Background and Aims:** Aberrant transcriptional programs are highly regulated processes that play important roles in the development and progression of hepatocellular carcinoma (HCC). Emerging evidence suggests that super-enhancers (SEs) often drive critical oncogene expression. However, SE-associated genes in HCC pathogenesis are still poorly understood.

**Methods:** We performed integrative ChIP-seq and Hi-C analyses of HCC cells and identified ajuba LIM protein (AJUBA) as a SE-associated gene. We evaluated AJUBA expression in HCC using immunohistochemistry, immunoblotting, and qRT-PCR. ChIP and luciferase reporter assays were performed to demonstrate that transcription factor 4 (TCF4) bound to AJUBA-associated SEs. We then assessed the role of AJUBA in HCC using both* in vitro* and *in vivo* assays. Epithelial-mesenchymal transition (EMT) was examined using immunofluorescence and immunoblotting assays. Furthermore, we used immunoprecipitation and BiFC assays to explore the underlying mechanisms.

**Results:** We identified AJUBA as a SE-associated oncogene in HCC regulated by TCF4. High AJUBA expression was related to an aggressive phenotype and unfavorable outcome in HCC patients. AJUBA knockdown significantly reduced cell migration and invasion capacities both* in vitro* and* in vivo*. Furthermore, AJUBA overexpression in HCC recruited tumor necrosis factor associated factor 6 (TRAF6), enhancing the phosphorylation of Akt and increasing Akt activity toward GSK-3β, thus promoting EMT.

**Conclusions:** Our results provide functional and mechanistic links between the SE-associated gene AJUBA and tumor EMT in aggressive HCC.

## Introduction

Hepatocellular carcinoma (HCC) is the most common primary liver malignancy and is a leading cause of cancer-related death worldwide 1. Most of HCC patients are not eligible for curative surgery due to the lack of early observable symptoms. In spite of the recent advances in surgical techniques and medical management, the long-term outcome of patients with HCC remains unsatisfactory because of the high incidence of recurrence and metastasis 2. Therefore, it is essential to clarify the molecular mechanisms underlying HCC recurrence and/or metastasis, as well as to identify new predictive biomarkers and novel therapeutic targets.

Metastasis is a primary cause of mortality in HCC and is triggered by a complicated succession of invasion-metastasis cascades 3. Epithelial-mesenchymal transition (EMT) is the initial process of tumor metastasis. It is a reversible process during which polarized epithelial cells change into detached mesenchymal cells through cytoskeleton rearrangement, resulting in decreased adhesion and increased cell motility, enhancing tumor dissemination 4. EMT is often triggered and controlled by various signaling pathways, such as TGF-β, Wnt, Notch, and PI3K/Akt 5. Additionally, EMT is also mediated by several transcription factors including Snail, Twist, Slug, and Zeb; among them, Snail has been found to be a major inducer of EMT 5, 6.

Interestingly, a recent study noted that super-enhancers (SEs) are implicated in specific tumor biology, including EMT 7. SEs, a large cluster of cis-regulatory DNA elements, can regulate cell-type specific genes by recruiting master transcription factors 8-10. SEs are often identified by histone markers, RNA polymerase 2 (Pol 2) and the transcription cofactor p300 11-13. During tumorigenesis, SEs frequently promote the transcription of prominent oncogenic genes in tumor cells. Importantly, SE-associated genes often have higher expression levels than those under the regulation of typical enhancers (TEs), which has been observed in a variety of cancers 13-15. The development of multiple cancers depends on SEs and SE-associated genes, making them promising therapeutic targets for human cancers. However, the roles of SEs and SE-associated genes in the development of HCC remain to be elucidated.

Here, we performed epigenomic analysis to characterize SE-associated genes in HCC cells. Integrative analysis identified ajuba LIM protein (AJUBA) as a novel HCC oncogene regulated by transcription factor 4 (TCF4), which is co-localized with the AJUBA promoter and AJUBA-SE. AJUBA functions as scaffolds and has been shown to modulate various biological events including cell proliferation, DNA damage response and differentiation 16-18. However, the role of AJUBA in cancers remains to be elucidated. In the present study, we further demonstrated that SE-driven AJUBA induces HCC cell EMT through the Akt/GSK-3β/Snail signaling pathway, ultimately leading to enhanced invasion and metastasis of HCC cells.

## Materials and Methods

### Human samples, tissue microarrays (TMAs), and cell lines

Paraffin-embedded pathological specimens from 168 patients with HCC were obtained from the archives of the Department of Pathology, Sun Yat-sen University Cancer Center in Guangzhou, China, between March 2008 and July 2015. The cases selected for this study had a distinctive pathological diagnosis of HCC, underwent primary and curative resection, had complete follow-up data, and were not receiving preoperative anti-cancer treatment. The median follow-up time was 48 months and the clinicopathological parameters are listed in Table 1. The TMAs were constructed according to a previously described protocol 19. We selected three cores of the sample from each tumor or adjacent liver tissue. Additionally, 32 pairs of fresh HCC and adjacent liver specimens were collected from the Biological Specimen Bank of Sun Yat-sen University Cancer Center. Samples used in this study were approved by the Medical Ethics Committee of Sun Yat-sen University Cancer Center.

HCC cell line SNU-449 was kindly provided by Dr. X.F. Steven Zheng (State Key Laboratory of Oncology in South China). The other HCC cell lines (SK-Hep1, Huh7, HepG2, Hep3B, MHCC97H and PLC/PRF/5) and human liver immortalized cells (MIHA and LO_2_) were kindly provided by Dr. Yun-Fei Yuan (Sun Yat-sen University Cancer Center). The cells were cultured in Dulbecco's Modified Eagle Medium (DMEM, Invitrogen, Carlsbad, CA, USA) or RPMI 1640 medium (Invitrogen, Carlsbad, CA, USA) supplemented with 10% fetal bovine serum (FBS, PAN-biotech, Germany) and 1% penicillin-streptomycin at 37 °C in an incubator containing 5% CO_2_. The Wnt3a-conditional medium (Wnt3a C.M.) was generated from the Wnt3a-expressing L cell line following the ATCC protocol.

### Immunohistochemistry (IHC) and cut-off value selection

TMA slides were incubated with anti-AJUBA antibody (1:60 dilution; Sigma-Aldrich, St. Louis, MO, USA). Immunostaining was performed using the Envision System with diaminobenzidine (Dako, Glostrup, Denmark), as previously described 20. Replacing the primary antibody with a normal IgG served as a negative control. The known positive cases were used as positive controls.

Nuclear immunoreactivity for AJUBA protein was scored in a semiquantitative way by evaluating the number of positive tumor cells over the total number of tumor cells. Scores were assigned in 5% increments (0%, 5%, 10% … 100%). The scores were assessed by two independent pathologists (MY Cai and D Xie) who were blinded to the clinicopathological data.

An X-tile plot was employed to assess AJUBA expression and optimize the cut-off value. The X-tile plots allowed us to determine an optimal cutoff value while correcting for the use of minimum P statistics by Miller-Siegmund P-value correction 21.

### Migration and invasion assays

Cell migration assay was performed using 8 μm transwell chambers (Falcon, USA). Cell invasion assay was performed with BD BioCoat Matrigel Invasion Chambers (Becton Dickinson Labware, Franklin Lakes, NJ) following the manufacturer's instructions. After 24 h incubation at 37 °C, the membranes were stained with 0.1% crystal violet for 15 min, the cells on the upper surface of the membrane were removed, and the cells on the lower side were then counted under a microscope. Both experiments were repeated in triplicate.

### Immunofluorescence (IF) analysis

Cells were fixed with 4% paraformaldehyde for 15 min at room temperature and then permeabilized with 0.2% Triton X-100 in phosphate buffered solution (PBS), blocked with 5% FBS in PBS for 1 h, and incubated with primary antibodies against vimentin (1:200 dilution, BD Transduction Laboratories, Franklin Lakes, New Jersey, USA) or β-catenin (1:200 dilution, BD Transduction Laboratories, Franklin Lakes, New Jersey, USA) overnight at 4 °C. After three washes, cells were incubated with an Alexa fluorescent-dye conjugated secondary antibody for 2 h. Finally, the nuclei were stained with DAPI (Beyotime, Shanghai, China) for 8 min, and the cells were imaged using fluorescence microscopy (LSM880 with fast Airyscan, ZEISS, Germany).

### Bimolecular fluorescence complementation (BiFC) assay

Tumor necrosis factor associated factor 6 (TRAF6) was cloned into the N-terminal half of YFP (YN-TRAF6) and AJUBA was fused to the C-terminal half of YFP (YC-AJUBA). YN-TRAF6 and YC-AJUBA were transfected individually or together into SNU-449 cells. YFP fluorescence was imaged 48 h after transfection with a laser scanning confocal microscope (LSM880 with fast Airyscan, ZEISS, Germany).

### *In vivo* mouse experiments

Experimental protocols and procedures involving mice were approved by the Institutional Animal Care and Use Committee of Sun Yat-sen University. Four-week-old Balb/c nude mice were purchased from Charles River Laboratories (Beijing, China). For the intravenous metastasis model, each mouse was injected with 2 × 10^6^ HCC cells via the tail vein. For the orthotopic implantation mouse model, mice were anesthetized with isoflurane and 2 × 10^6^ HCC cells suspended in 20 μL PBS containing 25% Matrigel (Corning, USA) were surgically implanted. Mice were sacrificed and examined for tumor nodules 8 weeks after implantation or vein injection.

### Co-Immunoprecipitation (co-IP)

For exogenous co-IP, whole-cell lysate was incubated with antibodies against Flag (1:200 dilution, Abmart, Shanghai, China) or Myc (1:200 dilution, MBL, Japan) on a rotary shaker overnight at 4 °C. The immunoprecipitate was then incubated with Sepharose-conjugated protein G magnetic beads (Thermo-Fisher Scientific, Waltham, MA, USA) at 4 °C for 5 h. The beads were then washed five times with TNE lysis buffer (10 mM Tris-HCl, 150 mM NaCl, 2 mM EDTA, and 0.5% Nonidet P-40, pH 7.5), resuspended in SDS sample buffer and boiled for 10 min.

For endogenous co-IP, cell lysate was incubated with anti-AJUBA (1:50 dilution, Santa Cruz Biotechnology, Santa Cruz, California, USA) antibody on a rotary shaker overnight at 4 °C. 30 μL of protein G magnetic beads were then added to the mixture and incubated at 4 °C for 5 h. The beads were washed five times and boiled to isolate the protein.

### Chromatin immunoprecipitation sequencing (ChIP-seq) and high throughput chromosome conformation capture (Hi-C) data analysis

ChIP-seq data of TCF4, Pol 2, H3K4me1, H3K4me3, and H3K27ac for HepG2 cells, and H3K27ac for Huh7 cells were obtained from the ENCODE database (https://www.encodeproject.org/) and re-analyzed using Romics. The Rank Ordering of Super Enhancers (ROSE) algorithm was used to compute and identify SEs from H3K27ac ChIP-seq data 9. Basically, enhancers within 12.5 kb were stitched together to define a single entity spanning a genomic region. The stitched and individual enhancers without neighboring enhancers within 12.5 kb were ranked by the level of H3K27ac signal in the genomic region. The stitched or individual enhancers with an H3K27ac signal above a cutoff where the slope of the distribution plot of H3K27ac ChIP-seq intensity is 1 were defined as SEs, and the remaining enhancers were considered TEs. The TCF4, H3K27ac, Pol 2, H3K4me1, and H3K4me3 bigwig files were visualized using the Integrative Genomics Viewer (IGV).

For Hi-C data analysis, raw Hi-C data (ENCBS061GMD and ENCBS232MEB) from the HepG2 cell line were downloaded from the ENCODE database. First, the raw data were filtered and trimmed to remove sequencing adaptors and low-quality reads. After quality control, clean reads were processed according to the Hi-Pro pipeline (Version 2.7.8) as previously described 22. The clean reads were mapped to the human genome (GRCh37, hg19) using Bowtie2 (http://bowtie-bio.sourceforge.net/bowtie2/index.shtml). After mapping, we generated raw contact matrices at a binning resolution of 40 kb, 100 kb, and 250 kb. The raw matrices were normalized using Iterative Correction and Eigenvector decomposition (ICE) to remove bias. Finally, the identified chromatin loops were visualized using the WASHU EPIGENOME BROWSER (http://epigenomegateway.wustl.edu/browser/).

### Dual-luciferase reporter assay

The AJUBA-SE was divided into five segments (E1-E5) and each segment was cloned into the pGL3-Basic plasmid (Additional file 2). SK-Hep1, HepG2, Huh7, SNU-449, and HEK293T cells were transfected with 1.2 μg of plasmids (pGL3-Basic, E1, E2, E3, E4, or E5) and 250 ng of pRL-TK (a normalization control) in 24-well dishes. Forty-eight hours after transfection, luciferase reporter activity was measured using the Dual-Luciferase Reporter Assay System (Vazyme Biotech, Nanjing, China). The ratio of firefly to renilla luciferase activity was normalized to the pGL3-Basic transfected cells. All assays were conducted independently in triplicate.

### ChIP-qPCR

The ChIP assay for TCF4 was performed using the EZ-Magna ChIP kit (EMD Millipore, Germany) according to the manufacturer's instructions. Briefly, HCC cell lysates were sheared via sonication to generate 200-500 bp fragments. Antibodies against TCF4 (1:50 dilution, Cell Signaling Technology, CA, USA) and rabbit-IgG (R&D Systems) were used for immunoprecipitation. Anti-rabbit IgG was used as a negative control. The ChIP products were quantified by qRT-PCR using specific primers to measure TCF4 enrichment.

### Statistical analysis

Statistical analysis was performed using SPSS 20.0 software (IBM, USA) and GraphPad Prism software (Version 7.0 for windows; GraphPad Prism Inc., San Diego, CA). Experimental data are presented as mean ± standard deviation (SD). Survival curves were obtained using Kaplan-Meier analysis and compared with log-rank tests. Univariate and multivariate survival analyses were performed using the Cox proportional hazards regression model. Pearson's correlation coefficients were determined in order to calculate the bivariate correlation between the studied variables. Student's *t* test was performed to analyze the difference between different groups. *P* < 0.05 was considered statistically significant (*P < 0.05, **P < 0.01, and ***P < 0.001).

## Results

### AJUBA is frequently upregulated in HCC and its expression correlates with prognosis

**Figure 1:**
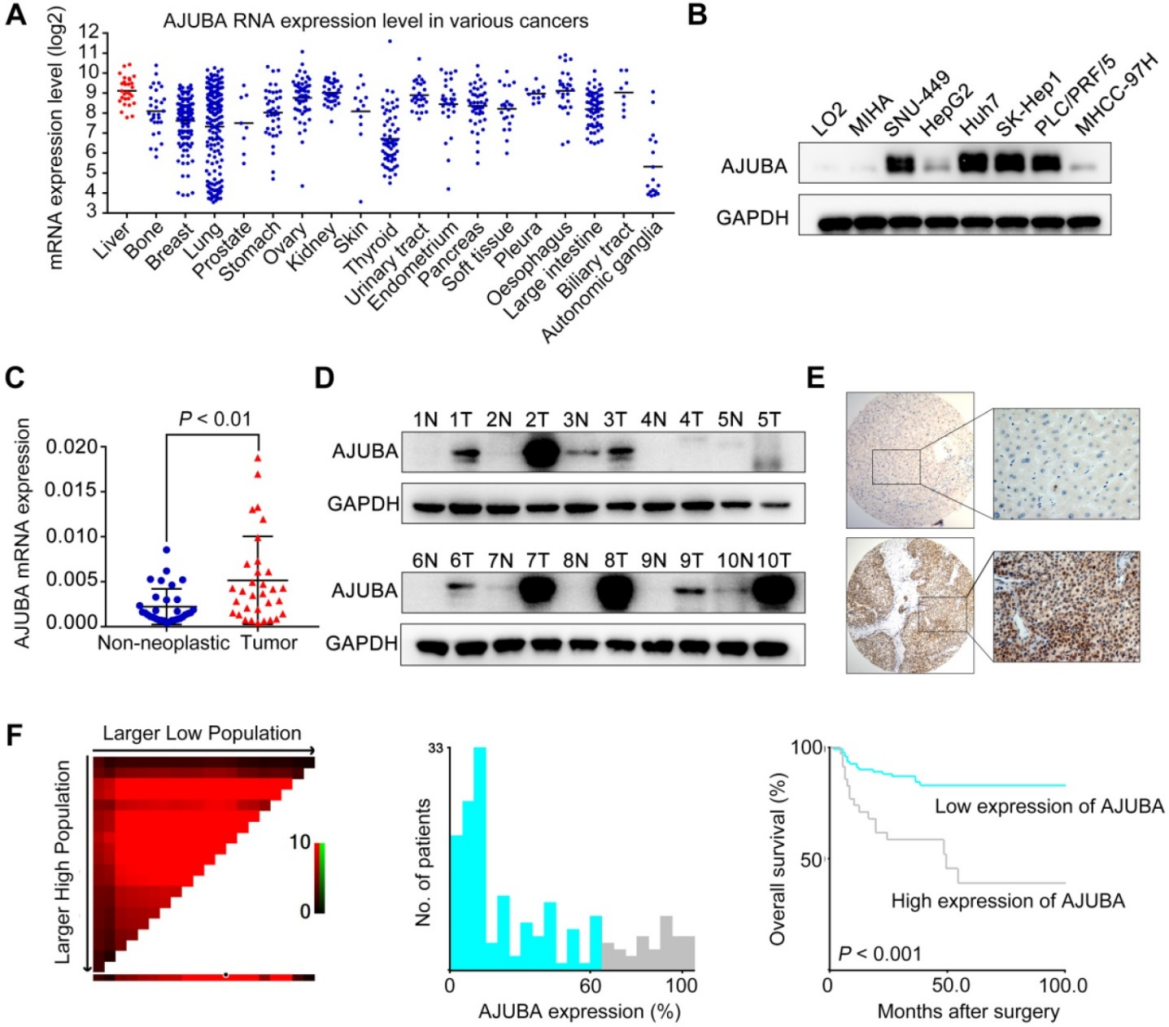
** AJUBA is upregulated in HCC.** (**A**) AUBA expression levels across various types of human cancer cell lines derived from the CCLE project from the Broad Institute. Each point represents one cell line. (**B**) Western blot analysis of AJUBA protein expression in 6 human HCC cell lines (SNU-449, HepG2, Huh7, SK-Hep1, PLC/PRF/5, and MHCC-97H) and 2 immortalized hepatocyte lines (LO_2_ and MIHA). GAPDH was used as a loading control. (**C**) Relative mRNA expression levels of AJUBA detected by qRT-PCR in 32 pairs of HCC and corresponding adjacent liver tissues. GAPDH was used to normalize the fold change. Dara are presented as the mean ± SD, n = 3. **P < 0.01. (**D**) Western blot analysis of AJUBA protein in 10 paired HCC and corresponding adjacent liver tissues. GAPDH served as a loading control. (**E**) Representative images showing negative AJUBA expression by IHC in a normal liver sample (upper panels) and high AJUBA expression in an HCC case (lower panels). (**F**) X-tile plots of the prognostic marker AJUBA. The cut-off point highlighted by the black/white circle in the left panel is shown on a histogram of the entire cohort (middle panel) and a Kaplan-Meier plot (right panel).

To explore the potential roles of SEs in HCC, we first re-analyzed publicly available ChIP-seq data for H3K27 acetylation (H3K27ac) in HepG2 and Huh7 cell lines from the ENCODE database. A total of 217 SE-associated genes in both cell lines were annotated (Figure S1A). Kyoto Encyclopedia of Genes and Genomes (KEGG) pathway analysis revealed that these 217 overlapping genes were significantly enriched in gene sets involved in cell adhesion and the TGF-β signaling pathway, suggesting that SEs may have important roles in EMT (Figure S1B). We then identified 23 (among the 217 candidates) up-regulated genes (Log_2_FC ≥ 0.5) that were correlated with poor overall survival (OS, *P* < 0.05) in TCGA HCC samples using the GEPIA web tool (Figure S1A and S1C-D). We first analyzed the Cancer Cell Line Encyclopedia (CCLE) project data and found that AJUBA is expressed at a relatively high level in HCC cells among various types of human cancer cells (Figure 1A). Thus, we chose AJUBA as a candidate target for further investigation. We also assessed AJUBA expression in a panel of immortalized hepatocyte and HCC cell lines. AJUBA expression was significantly higher in HCC cell lines compared to the immortalized LO_2_ and MIHA hepatocyte lines (Figure 1B). Moreover, AJUBA mRNA and protein levels in HCC tissue samples were higher than in adjacent nonmalignant liver tissues derived from SYSUCC patients (Figure 1C -D).

To investigate the expression dynamics of AJUBA in HCC, we performed AJUBA immunostaining on an HCC TMA containing 168 pairs of HCC specimens and the corresponding normal liver tissues from SYSUCC cohort. The representative images of AJUBA staining in HCC and normal liver tissue samples are shown in Figure 1E. We divided the HCC cohort into low and high populations based on a cutoff value (determined using the X-tile program) of more than 60% AJUBA positive cells (Figure 1F). Correlation analysis showed that high AJUBA expression was positively associated with tumor multiplicity, poor differentiation, advanced TNM stage, the presence of vascular invasion, and tumor relapse (Table 1). Kaplan-Meier analysis demonstrated that AJUBA expression was significantly correlated with poor patient survival (Figure 1F). Further, multivariate Cox regression analysis showed that AJUBA expression was an independent prognostic factor for poor survival of HCC patients (relative risk: 2.189, confidence interval: 1.058 - 4.529, *p* = 0.035) (Table 2). These findings suggest that a potential function of AJUBA is closely related to HCC tumorigenesis and progression.

### AJUBA is a SE-associated gene in HCC

**Figure 2:**
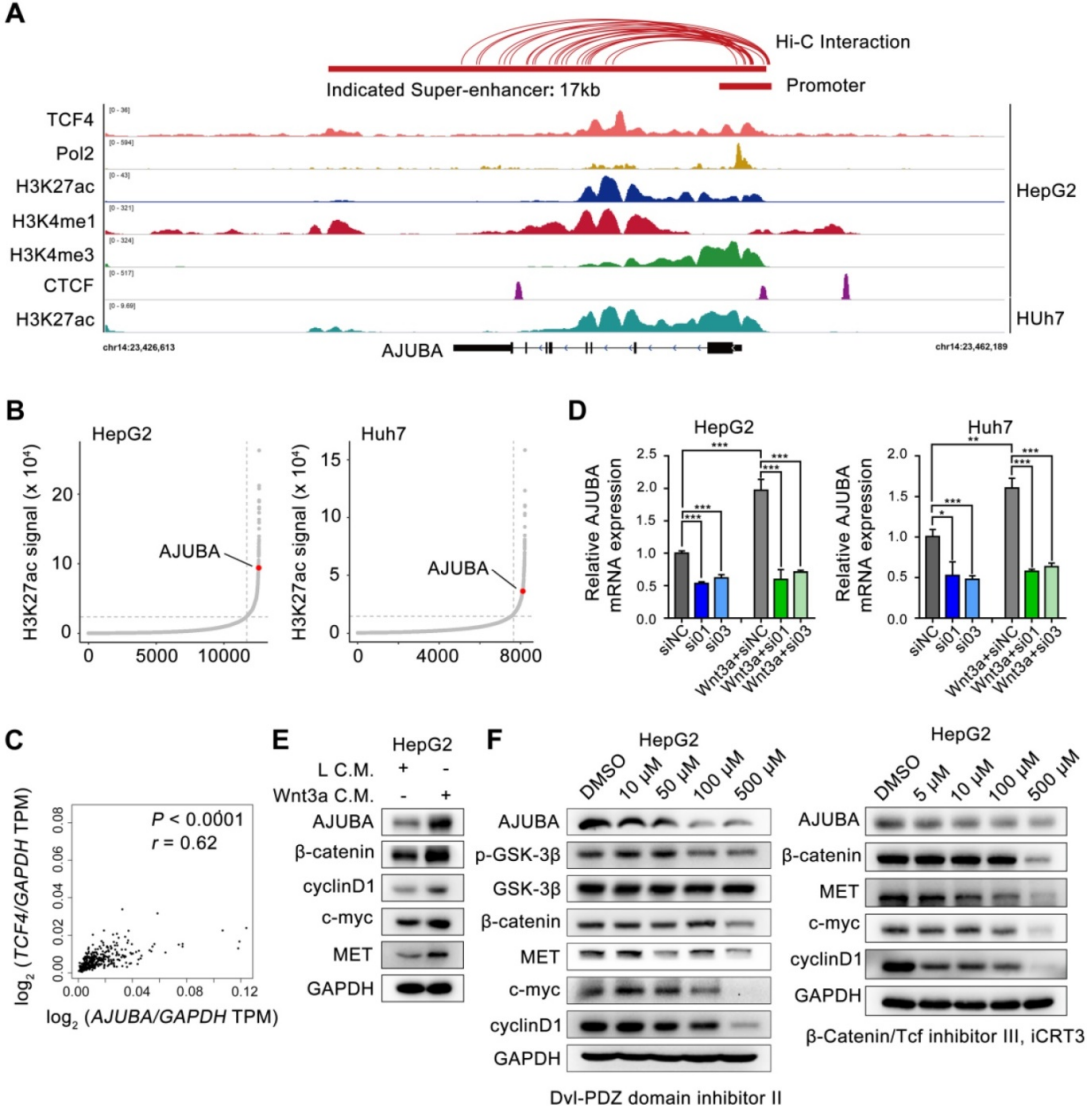
** AJUBA is a super-enhancer driven gene in HCC.** (**A**) ChIP-seq profiles for Pol 2, H3K27ac, H3K4me1, H3K4me3, and TCF4 at the AJUBA locus in HepG2 cells, as well as H3K27ac in Huh7 cells. Predicted SE and promoter are depicted as red bars. Above the ChIP-seq profiles are interactions between SE and promoter regions in HepG2 cells predicted by Hi-C from ENCODE database, and visualized using WashU Epigenome Browser. (**B**) Hockey stick plots on the basis of their input-normalized H3K27ac signals in HepG2 and Huh7 cells. SE-associated AJUBA is highlighted. (**C**) The correlation of TCF4 and AJUBA mRNA levels in HCC tissues. TCGA data were analyzed using the GEPIA web tool. Correlation between TCF4 and AJUBA was determined by Pearson's rank correlation coefficient. *r,* Pearson correlation coefficient; *P,* Pearson *p* value. (**D**) The relative expression levels of AJUBA mRNA after silencing TCF4 in three HCC cell lines with or without Wnt3a C.M. stimulation overnight. Quantitative data are presented as the mean ± SD. **P < 0.01, ***P < 0.001. (**E**) The expression of AJUBA and Wnt pathway components in HepG2 cells treated with either L C.M. or Wnt3a C.M. for 24 h. (**F**) The protein levels of AJUBA and Wnt pathway components in HepG2 cells treated with different Wnt signaling inhibitors.

To gain mechanistic insight into the possible transcriptional mechanism of AJUBA in HCC tissues, we re-analyzed publicly available H3K27ac ChIP-seq data and identified a 17 kb SE overlap with AJUBA, which was also confirmed by markers such as monomethylation of the 4th lysine of histone 3 (H3K4me1) and Pol 2 (Figure 2A). SE-associated AJUBA was annotated in HepG2 and Huh7 cells (Figure 2B). We then used JQ1, a BRD4 inhibitor, to disrupt the activity of SEs, which has been proven effective in a variety of cancers 23-26. Notably, AJUBA expression was significantly downregulated in HCC cells treated with JQ1 (Figure S2A-B). Next, by re-analyzing the publicly available Hi-C data of HepG2 cells, we determined the extensive interactions between putative SE and the AJUBA promoter region (≈2 kb). Meanwhile re-analysis result of CTCF ChIP-seq data showed a DNA-loop between them (marked with red connecting lines above the ChIP-seq profiles) in Figure 2A 27, 28. Since the Wnt signaling pathway members are mutated in 44% of HCC cases 29, we next explored whether TCF4 regulated the expression of AJUBA. We first analyzed the correlation between AJUBA protein expression and TCF4 mRNA, and we found that mRNA level of TCF4 was positively correlated with AJUBA expression (Figure 2C). We then transfected siRNAs targeting TCF4 in HCC cells, and we found knockdown of TCF4 resulted in significant inhibition of AJUBA (Figure S2C-D and Figure 2D). Additionally, we found that AJUBA mRNA expression was induced by Wnt3a, which was significantly inhibited after depletion of TCF4 (Figure 2D). The expression levels of Wnt pathway targets, including cyclin D1, c-myc, and MET, were also enhanced by Wnt3a (Figure 2E and Figure S2E). Finally, we observed the effects of three Wnt inhibitors on AJUBA, including Dvl-PDZ domain inhibitor II (which blocks Dvl2 binding to LRP6), lithium (which suppresses GSK3β), and iCRT3 (which disrupts the interactions between β-catenin and TCF4) (Figure 2F and Figure S2E). These results demonstrate that in HCC cells, Wnt signaling induces expression of AJUBA as well as some other Wnt targets. Taken together, these results indicate that SE-associated AJUBA is a downstream of TCF4.

### TCF4 activates AJUBA transcription by binding to its SE

**Figure 3:**
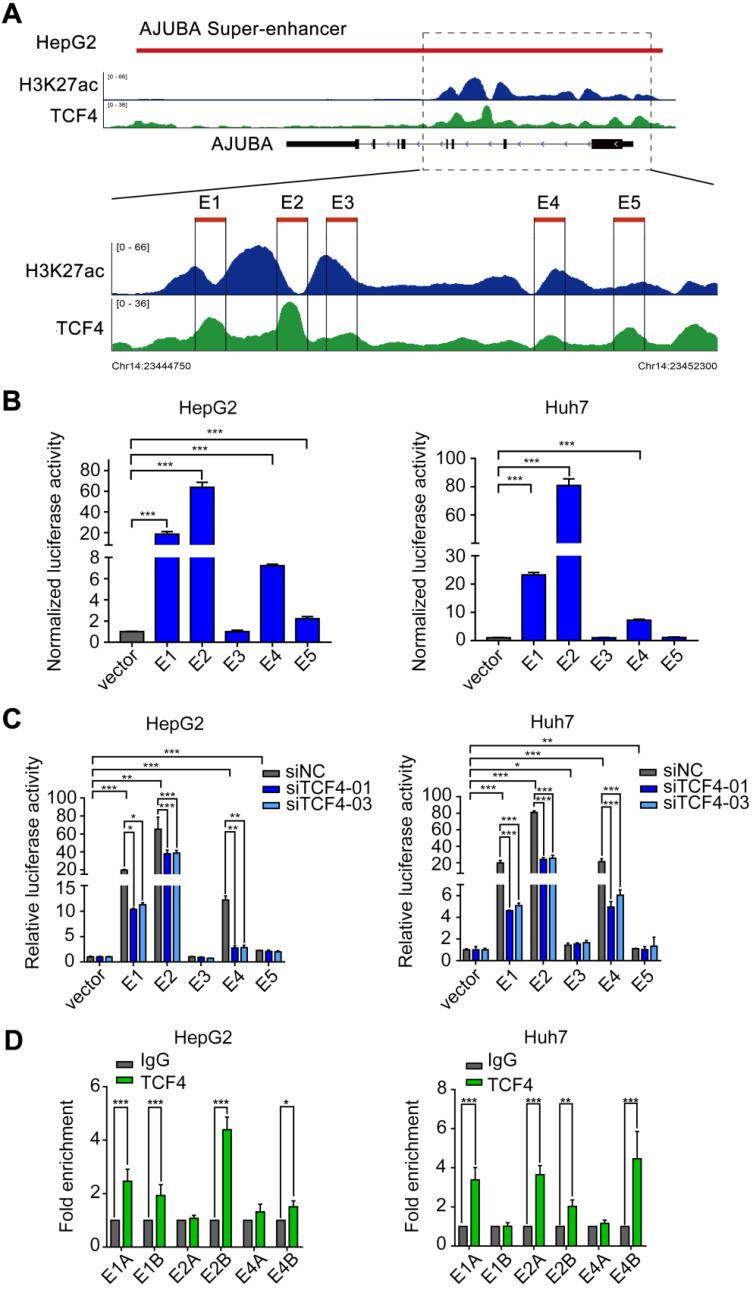
** TCF4 activates AJUBA transcription by binding to the AJUBA super-enhancer.** (**A**) H3K27ac and TCF4 ChIP-seq signals at the AJUBA locus in HepG2 cells. Five TCF4-specific elements (E1-E5) within the SE were separately cloned into the luciferase reporter vector pGL3-Basic. (**B**) Enhancer activity measured by dual-luciferase reporter assay in HepG2 and Huh7 cells. (**C**) The luciferase activities of each of the five enhancer elements measured in HepG2 and Huh7 cells after silencing TCF4. For (B and C), luciferase signal was normalized to a renilla transfection control. (**D**) ChIP-qPCR analysis of the interaction between TCF4 and segments of E1, E2, and E4 (E1A, E1B, E2A, E2B, E4A, and E4B) in HepG2 and Huh7 cells. Rabbit normal IgG antibody was used as a negative control. Data in B-D are representative of 2-3 separate experiments. *P < 0.05, **P < 0.01, ***P < 0.001.

Since SEs often recruit a high density of transcription factors and coactivators to drive oncogene expression in tumor cells 9, 30, 31, we hypothesized that TCF4 might be responsible for maintaining AJUBA-SE activity. To this end, we first re-analyzed the publicly available TCF4 ChIP-seq data and revealed several prominent peaks in the putative SE region of AJUBA (Figure 2A and Figure 3A). We determined the enriched region of TCF4 in AJUBA-SE as the TCF4-specific elements. To obtain clues to that TCF4 contributes to the activity of AJUBA-SE, we cloned five TCF4-specific elements (E1-E5) into the pGL3-Basic plasmid and measured their activities using luciferase reporter assay. E1, E2, and E4 had significantly higher enhancer activities in all HCC cells compared with the empty control vector, but not in HEK293T cells (Figure 3B and Figure S3B). Importantly, silencing TCF4 in HCC cells reduced the enhancer activity of E1, E2, and E4 (Figure 3C and Figure S3C). Furthermore, the enrichment of TCF4 in these elements was confirmed by ChIP-qPCR (Figure 3D, Figure S3A and S3D-E). Finally, we used CRISPR/Cas9 editing to delete the genomic region containing E2 (Figure S4A-B). The deletion suppressed AJUBA protein level relative to a non-edited control (Figure S4C). Taken together, these results suggest that AJUBA is tightly regulated by the interaction between TCF4 and AJUBA-SE.

### AJUBA promotes HCC cell migration and invasion ability both *in vitro* and *in vivo*

**Figure 4:**
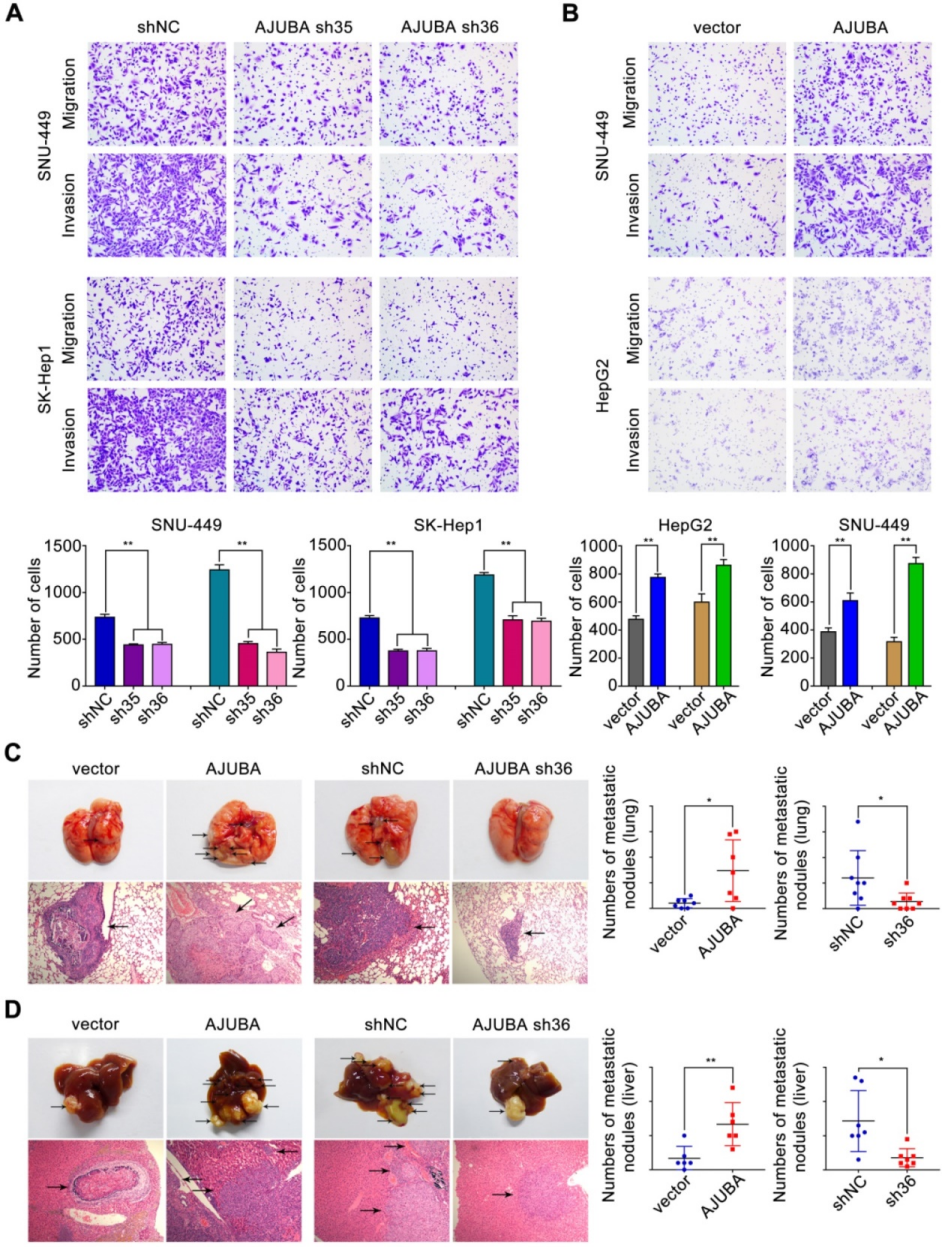
** AJUBA increases HCC cell migration and invasion both *in vitro* and *in vivo*.** (**A-B**) Transwell migration and matrigel invasion assays were performed in cells with AJUBA knockdown or overexpression. The bottom panel shows bar charts quantifying cell migration and invasion. (**C**) A murine tail vein injection lung metastasis model. Representative images of gross lung metastatic foci and H&E histology (original magnification: 200×) are shown in the left panel. The right panel shows graphs of the number of metastatic nodules formed in the lungs of mice 8 weeks after injection of HepG2-vector, HepG2-AJUBA (n = 7/group), SK-Hep1-shNC, and SK-hep1-sh36 transfected cells (n = 8/group), respectively. (**D**) An orthotopic implanted intrahepatic metastasis model. Representative images of gross intrahepatic metastasis foci and H&E histology (original magnification: 200×) of hepatic metastases are shown in the left panel. The scatter grams (right panel) show the number of metastatic nodules formed in the livers of mice 8 weeks after orthotopic implantation with HepG2-vector, HepG2-AJUBA (n = 6/group), SK-Hep1-shNC, and SK-hep1-sh36 transfected cells (n = 7/group), respectively. Statistical results are presented as the mean ± SD. *P < 0.05, **P < 0.01.

To identify the functional role of AJUBA in HCC cells, we used SK-Hep1 cells with high AJUBA expression for loss-of-function and HepG2 cells with low AJUBA expression for gain-of-function assays. Meanwhile, SNU-449 cells displaying moderate expression level of AJUBA were used for both loss-of-function and gain-of-function assays. Using migration and invasion assays, we found that cell migration and invasion were attenuated after AJUBA knockdown in SNU-449 and SK-Hep1 cells (Figure 4A), which was also confirmed by AJUBA-knockdown Huh7 cells (Figure S5B). In contrast, AJUBA overexpression enhanced migration and invasion in HepG2 and SNU-449 cells compared to the negative control (Figure 4B). These results were further confirmed in a wound healing assay (Figure S5A).

In order to assess the effect of AJUBA *in vivo*, stably transfected cell lines (HepG2-vector, HepG2-AJUBA, SK-Hep1-shNC, and SK-Hep1-sh36) were injected into the lateral tail vein of nude mice. Eight weeks after injection we found a significant increase in the number and size of lung metastasis nodules in mice injected with HepG2-AJUBA cells compared to controls. The group injected with SK-Hep1 cells lacking AJUBA had remarkably decreased metastatic nodules in the lung compared to controls. This was further confirmed by haematoxylin and eosin (H&E) staining of dissected lungs (Figure 4C). Furthermore, we transplanted tumor cells into mouse liver to establish a liver orthotopic model. Eight weeks after transplantation, we evaluated intrahepatic metastases tumor nodules and found more tumor nodules in the HepG2-AJUBA group compared to controls (Figure 4D). Taken together, these results suggest that AJUBA may be involved in the aggressive phenotype of HCC cells both* in vitro* and *in vivo*.

### AJUBA promotes EMT in HCC

**Figure 5:**
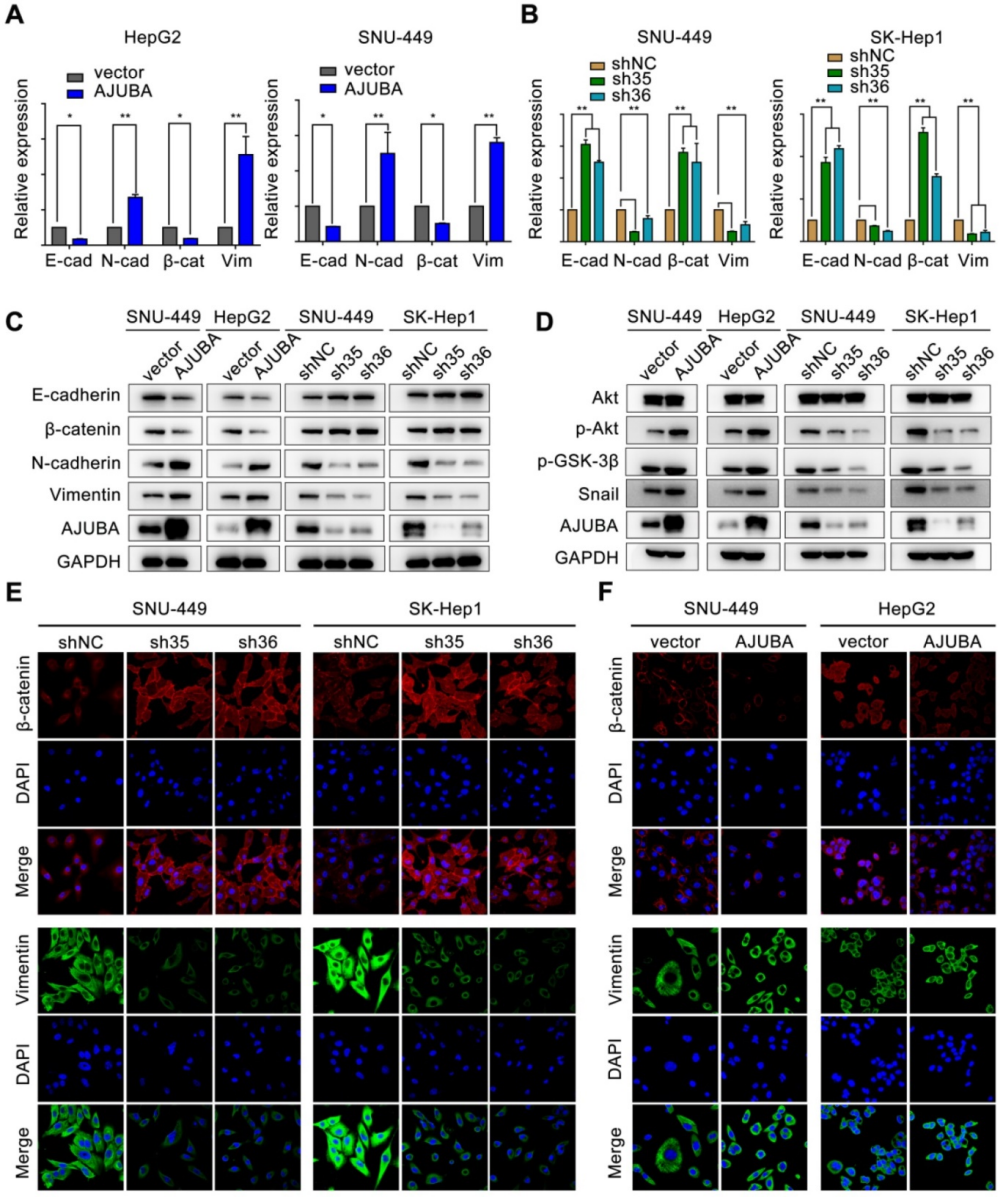
** AJUBA promotes EMT in HCC.** The relative mRNA (**A, B**) and protein (**C**) expression levels of EMT markers (E-cadherin, β-catenin, N-cadherin, and vimentin) in AJUBA-overexpressed and AJUBA-silenced cells. The results shown in (A) and (B) are representative of three independent experiments. All error bars show standard error of the mean. *P < 0.05 and **P < 0.01. (**D**) The expression levels of Akt, p-Akt, p-GSK-3β, Snail, and GAPDH (loading control) measured by western blotting. (**E-F**) IF staining of β-catenin and vimentin in the indicated cell lines. Nuclei were counterstained with DAPI (magnification: 600×).

EMT is a crucial cellular process associated with tumor initiation, invasion, and metastasis in which cells lose epithelial features and acquire mesenchymal characteristics 32. We next sought to evaluate the effect of AJUBA on EMT. We found that AJUBA-overexpressed HCC cells exhibited a scattering and elongating phenomenon; however, AJUBA knockdown cells turned to relative round morphology (Figure S5D). Real-time PCR and western blotting assays demonstrated that ectopic AJUBA expression decreased the levels of epithelial markers (E-cadherin and β-catenin), but increased the levels of mesenchymal features (N-cadherin and vimentin). The opposite effect was observed in AJUBA-depleted cells (Figure 5A-C and Figure S5C). Similar results were also observed in IF staining assays. Knockdown of AJUBA increased β-catenin expression while decreased vimentin expression in SNU-449 and SK-Hep1 cells (Figure 5E). In contrast, overexpression of AJUBA decreased the epithelial marker β-catenin and increased the mesenchymal marker vimentin (Figure 5F). These findings indicate that AJUBA promotes EMT in HCC cells.

### AJUBA induces EMT by activating the Akt/GSK-3β/Snail pathway in HCC

**Figure 6:**
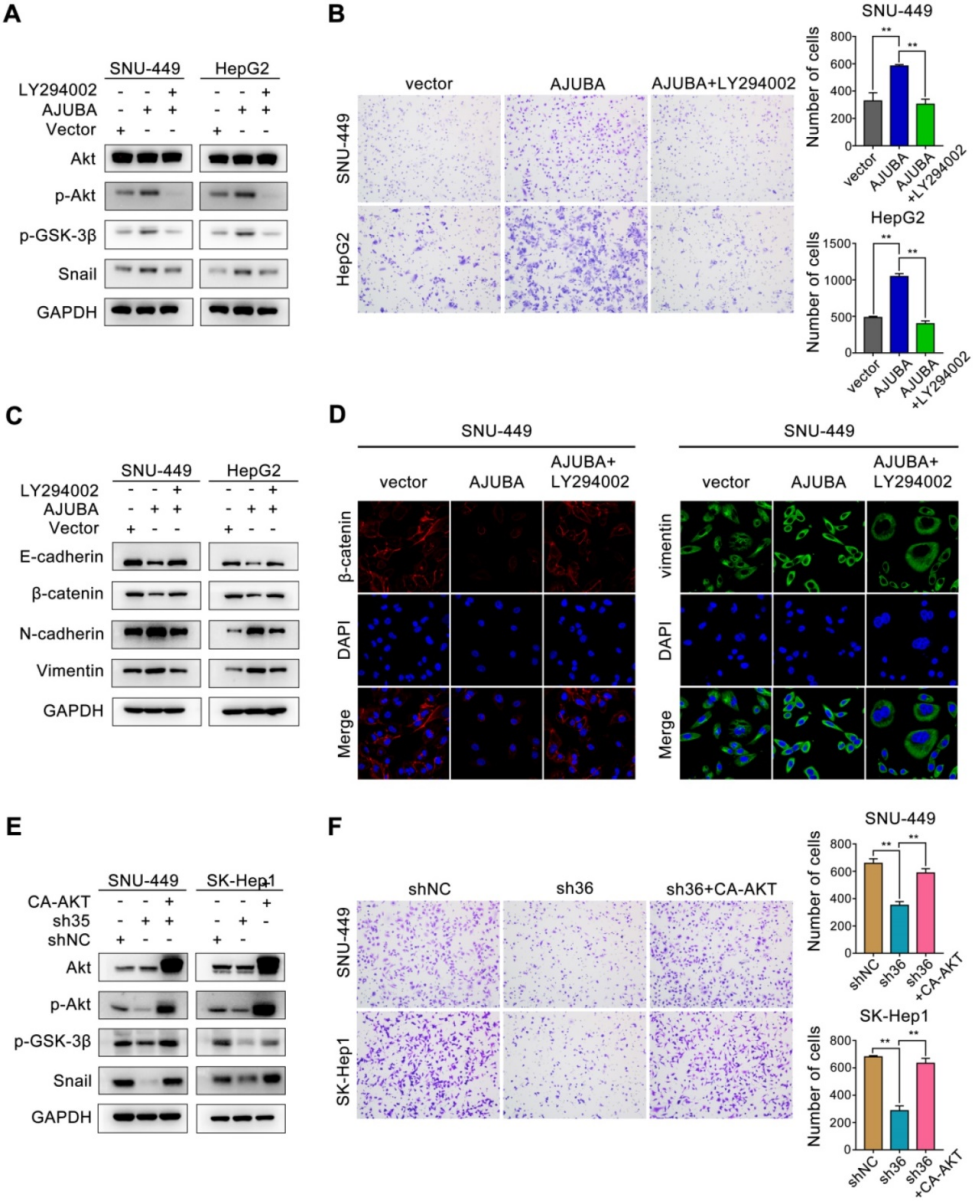
** The Akt/GSK-3β/Snail pathway is responsible for AJUBA-induced EMT and invasiveness.** (**A**) Western blotting demonstrated that the Akt inhibitor LY294002 could effectively decrease p-Akt, p-GSK-3β, and Snail expression induced by AJUBA. (**B**) A transwell migration assay showed that LY294002 could effectively inhibit AJUBA induced cell migration. The right panels show bar charts of cell migration quantification. Three independent experiments were performed. Western blots (**C**) and IF images (**D**) showed that AJUBA-induced increase in mesenchymal marker expression and decrease in epithelial marker expression could be rescued by LY294002 treatment. (**E**) Western blotting demonstrated that CA-AKT transfection effectively increased p-Akt, p-GSK-3β, and Snail expression after AJUBA silencing. (**F**) A transwell migration assay showed that CA-AKT could effectively promote cell migration ability after AJUBA silencing. The right panel shows bar charts of cell migration quantification. Three independent experiments were performed. All error bars show standard error of the mean. **P < 0.01.

We next sought to obtain mechanistic insight into how AJUBA promotes EMT in HCC cells. Of note, a multitude of signaling pathways such as TGF-β/Smad 33, PI3K-Akt 34, Wnt/β-catenin 35, and p53 36, as well as many transcription factors 37, have been identified to be related to the EMT process. Snail is a predominant transcription factor frequently upregulated by diverse inducers in various cancers 38. Inhibition of GSK-3β by activating the PI3K-Akt signaling pathway improves the stability and nuclear localization of Snail, which induces EMT 39. Thus, we examined the Akt/GSK-3β/Snail signaling pathway after AJUBA overexpression, and we found that the levels of phosphorylated Akt (p-Akt), phosphorylated GSK-3β (p-GSK-3β), and Snail increased in SNU-449 and HepG2 cells. Consistently, opposite changes were detected after silencing AJUBA in SNU-449 and SK-Hep1 cells (Figure 5D). To further confirm these results, we used the PI3K inhibitor LY294002 to further explore the relationship between decreased p-Akt and increased cell migration induced by AJUBA in SNU-449-AJUBA and HepG2-AJUBA cells, and we found PI3K inhibition effectively decreased the levels of p-Akt, p-GSK-3β and Snail, which could be reversed by overexpression of AJUBA (Figure 6A). Transwell assay also demonstrated that LY294002 could impair the increased cell migration induced by ectopic AJUBA expression (Figure 6B). Western blotting analysis showed that PI3K inhibition disrupted mesenchymal features (N-cadherin and vimentin) and increased the levels of epithelial markers (E-cadherin and β-catenin) in SNU-449-AJUBA and HepG2-AJUBA cells (Figure 6C). Similar results were also observed using IF assay (Figure 6D). We next transfected AJUBA-silenced cells with constitutively active Akt gene (CA-AKT) to further investigate the effect of AJUBA on the Akt/GSK-3β/Snail pathway. As expected, the decreased expression of p-Akt, p-GSK-3β, and Snail observed after silencing AJUBA was significantly rescued by CA-AKT (Figure 6E, Figure S7A and S7C). Further, the attenuated migration capacity could also be rescued by overexpression of CA-AKT as indicated in transwell assay (Figure 6F). Collectively, these findings demonstrate that the Akt/GSK-3β/Snail signaling pathway is at least partially responsible for AJUBA-induced EMT and invasiveness in HCC cells.

### TRAF6 is responsible for AJUBA-activation of the Akt/GSK-3β/Snail pathway

**Figure 7:**
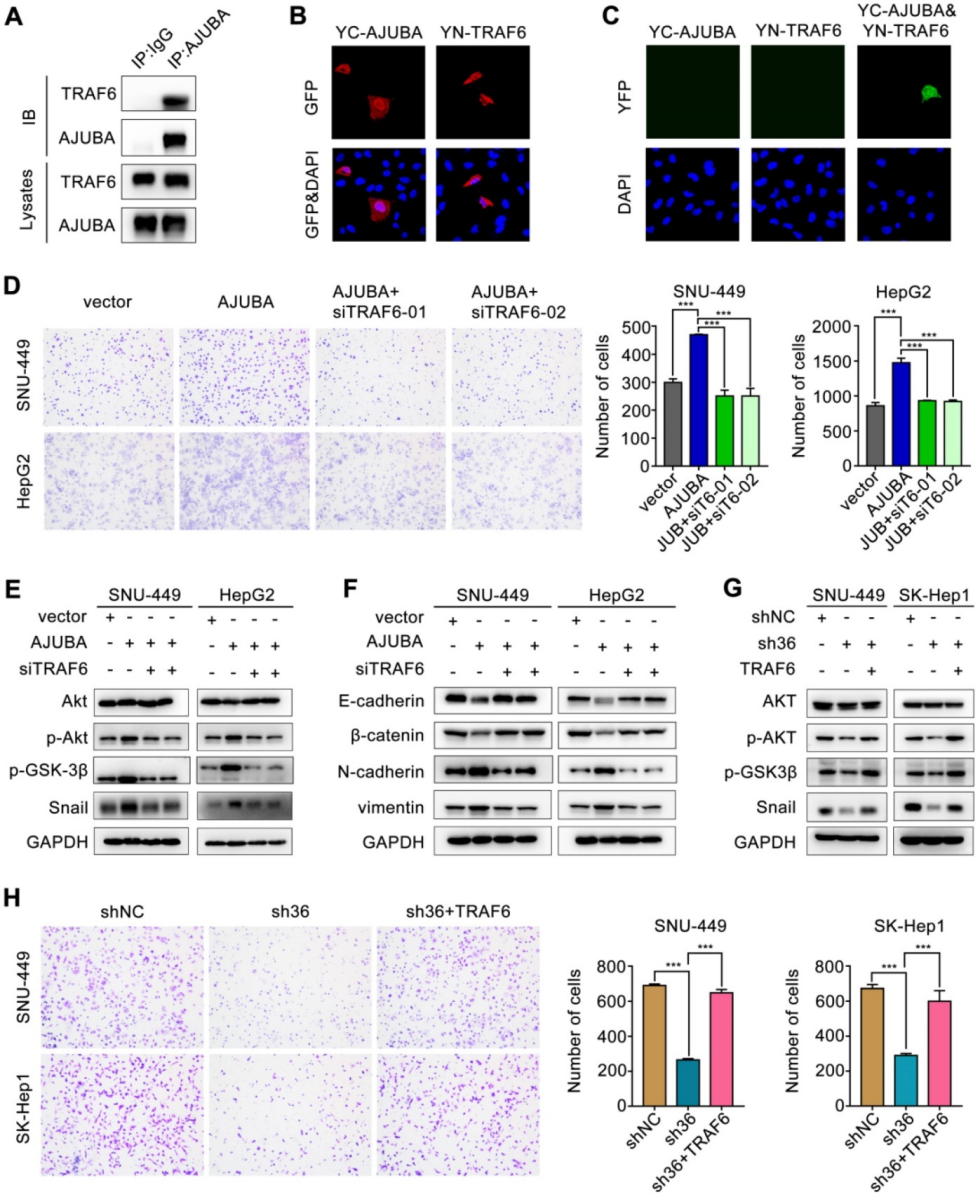
** TRAF6 is involved in the aggressive AJUBA-induced phenotype.** (**A**) Overexpressed AJUBA interacts with endogenous TRAF6 demonstrated by co-IP in HepG2-AJUBA cells. (**B-C**) Detection of the AJUBA-TRAF6 interaction in living cells with BiFC assay. YC-AJUBA and YN-TRAF6 were transfected alone or together into SNU-449 cells. The expression of YC-AJUBA and YN-TRAF6 was detected with an anti-GFP antibody. The reconstituted YFP fluorescence was detected at 488 nm. (**D**) A transwell migration assay showed that AJUBA overexpression promoted cell migration, which could be reversed by TRAF6 silencing. The right panels show bar charts of cell migration quantification. Three independent experiments were performed. (**E**) Western blotting demonstrated that silencing TRAF6 effectively decreased AJUBA-induced p-Akt, p-GSK-3β, and Snail expression. (**F**) The decreased expression of epithelial markers (E-cadherin and β-catenin) and increased expression of mesenchymal markers (N-cadherin and vimentin) were reversed after silencing TRAF6. (**G**) p-Akt, p-GSK-3β, and Snail expression, which were decreased AJUBA knockdown, were effectively rescued by TRAF6 upregulation. (**H**) A transwell migration assay showed that silencing AJUBA inhibited cell migration, which could be reversed by TRAF6 upregulation. The right panels show bar charts of cell migration quantification. Three independent experiments were performed. All error bars show standard error of the mean. ***P < 0.001.

Previous studies showed that TRAF6 could promote the K63-linked ubiquitination of Akt, thus suppressing GSK-3β and stabilizing Snail 40, 41. Since AJUBA could modulate IL-1-induced NF-κB activation by interacting with TRAF6 42, we hypothesized that TRAF6 might be involved in AJUBA-induced aggressive HCC phenotype. To address this, we performed a co-IP assay with an anti-AJUBA antibody in HepG2-AJUBA cells and found that AJUBA and TRAF6 form a complex *in vivo* (Figure 7A). Consistent with this, we performed exogenous co-IP assay by overexpressing epitope-tagged AJUBA and TRAF6 in HEK293T cells, further demonstrating a physical interaction between them (Figure S6A). Moreover, the expression level of TRAF6 was positively correlated with AJUBA mRNA level (Figure S6B). Cell fractionation and confocal microscopy assays showed that AJUBA and TRAF6 were mainly located in the nucleus and cytoplasm (Figure 7B and Figure S6C). Thus, we speculated that the AJUBA-TRAF6 interaction had the same cellular distribution. To confirm these, we performed a BiFC assay in SNU-449 cells. The reconstituted fluorescent YFP from YC-AJUBA (fusion of AJUBA to the C-terminal half of YFP) and YN-TRAF6 (fusion of the N-terminal half of YFP to TRAF6) was observed in both the nuclei and cytosol (Figure 7C). These observations strongly support the idea that AJUBA is associated with TRAF6 in HCC.

Next we performed ubiquitination assay by co-transfecting SNU-449 stable cell lines with HA-ubiquitin (K63 only), S-tag-AKT and Flag-TRAF6 in the overexpression or knockdown of AJUBA. Our results showed that ectopic expression of AJUBA increased the K63 ubiquitination of Akt (Figure S6D). In contrast, the K63 ubiquitination of Akt significantly decreased in SNU-449 cells with downregulated AJUBA (Figure S6E). As a result, we sought to confirm the oncogenic function of AJUBA via interacting with TRAF6 to activate the Akt/GSK-3β/Snail pathway. We disrupted TRAF6 in SNU-449-AJUBA and HepG2-AJUBA cells by introducing a siRNA. The efficiency of siTRAF6 was validated in SNU-449 cells (Figure S6F-G). The increased cell migration induced by overexpression of AJUBA was rescued after TRAF6 knockdown as indicated in transwell assay (Figure 7D). We next examined the expression levels of several members involved in the Akt/GSK-3β/Snail pathway using western blotting. We observed that the increased expression of p-Akt, p-GSK-3β, and Snail induced by AJUBA was effectively decreased by siTRAF6 (Figure 7E). Furthermore, we measured the expression of EMT-related markers following siTRAF6, and we found their changes were consistent with an alteration in the Akt/GSK-3β/Snail pathway (Figure 7F). While TRAF6 overexpression generated the opposite effects (Figure 7G-H, Figure S6H-I, S7B and S7D). These results suggest that TRAF6 is responsible for AJUBA-induced aggressive HCC phenotype via the Akt/GSK-3β/Snail pathway.

## Discussion

**Figure 8:**
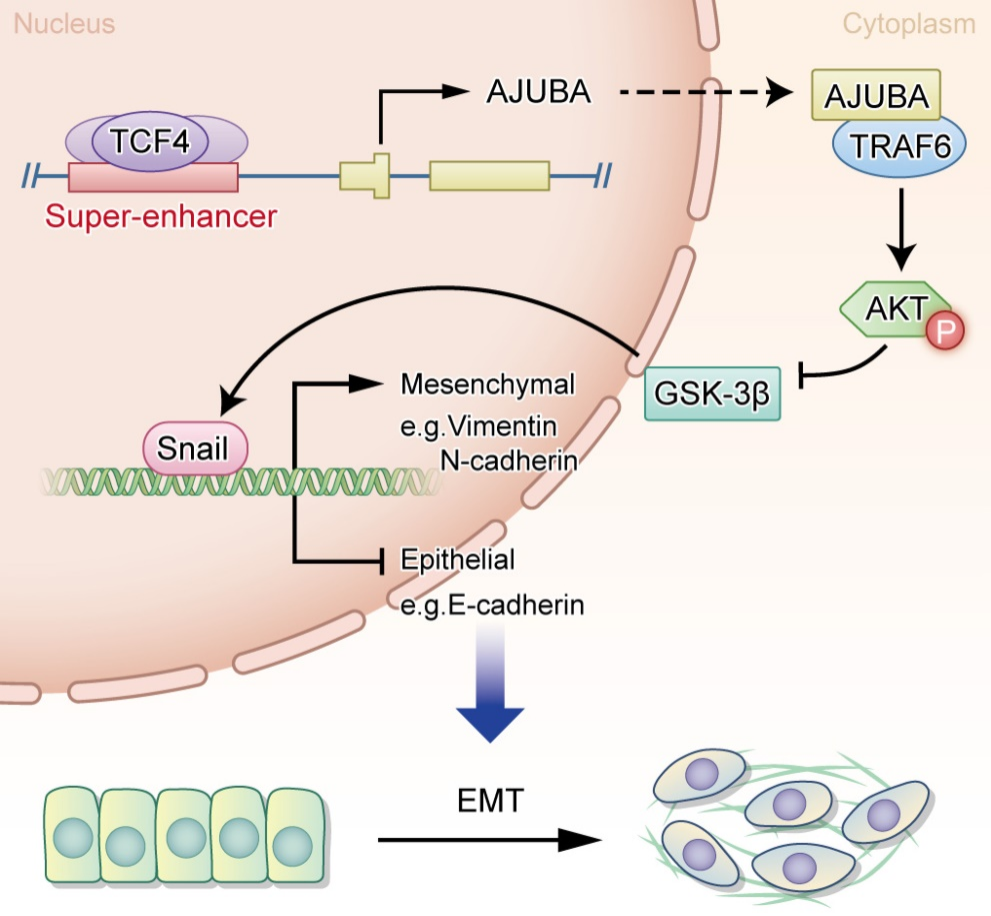
** A proposed working model of transcriptional regulation in HCC biology.** The transcription factor TCF4 binds to the AJUBA promoter and SE regions, drives its expression, and leads to the upregulation of AJUBA in HCC, thus promoting EMT through the activation of the Akt/GSK-3β/Snail pathway.

In the current study, we provide evidence showing that AJUBA is upregulated in HCC tissues and its high expression is related to a more aggressive malignancy and adverse clinical outcome. Moreover, our data support a crucial role for AJUBA in promoting EMT of HCC cells via the Akt/GSK-3β/Snail signaling pathway. Interestingly, AJUBA up-regulation in HCC was found to be mediated by the direct binding of TCF4 to its SE and promoter region. Our findings provide functional and mechanistic links between AJUBA and HCC EMT (Figure 8).

HCC outcomes remain dismal because of its high recurrence and/or metastasis rates. Therefore, understanding the genetic and epigenetic alterations driving the pathogenesis of tumor recurrence and/or metastasis is of great importance. EMT plays a key role in tumor invasion and metastasis, and it has been shown to contribute to HCC progression 43. Recently, multiple transcription factors and genes have been reported to be involved in EMT 44-47. However, the functions of SEs and SE-associated genes in EMT remain poorly understood. In this study, we performed an integrative analysis of both ChIP-seq and Hi-C data and identified a 17 kb SE interacting with AJUBA. We then demonstrated that high AJUBA expression in HCC was important in the acquisition of an aggressive and/or poor prognostic phenotype. In addition, AJUBA expression was found to decrease the RNA and protein levels of epithelial markers and increase mesenchymal features in HCC cells. Furthermore, key EMT transcription factor Snail, AJUBA, and PRMT5 ternary complex can be found at the proximal promoter region of E-cadherin, repressing E-cadherin expression and regulating EMT signaling 48. Collectively, these results suggest that super-enhancer associated AJUBA is highly relevant to HCC cell EMT and aggressiveness.

A series of studies have indicated that master transcription factors, frequently localized at SEs, mediate transcriptional dysregulation and promote tumorigenesis in squamous cell carcinoma 49, as well as the progression of esophageal squamous cell carcinoma 50 and Ewing sarcoma 27. However, whether SE can regulate the expression of oncogenes in HCC through its interaction with key transcription factors remains to be elucidated. Recent studies have found abnormal activation of the Wnt/β-catenin signaling axis in HCC carcinogenicity and that 44% of HCCs display gene alterations in the Wnt pathway, of which CTNNB1 mutation is increased up to 35% 29, 51. Moreover, as a co-transcription factor of TCF4, β-catenin gene mutation and protein accumulation lead to the aberrant activation of multiple TCF4 target genes in HCC 52, 53. These findings suggest that TCF4 may serve as a key transcription factor in HCC cells. What's more, TP53 mutation also plays important role in HCC, so we detected the correlation of mutant TP53 and AJUBA 29. But there was no difference in the expression of AJUBA under different TP53 stated in the patients from TCGA (data not shown).

Remarkably, SEs usually bind large numbers of master transcription factors that define cell identity, and thus participate in a variety of pathophysiological processes 9, 23, 30. Recent studies have demonstrated several transcription factors that directly bind to SEs to activate the transcription of EMT-related genes in HCC 7, 54. A functional association between the Wnt signaling pathway and EMT has also been well established. Nevertheless, how SEs regulate EMT through transcriptional regulation of the Wnt pathway remains unclear. In our study, we found that AJUBA overexpression in HCC is regulated by TCF4 directly binding to its promoter and SE region, activating AJUBA transcription and promoting EMT in HCC cells. These findings imply that the interaction of AJUBA-SE and TCF4 is very important in the activation of EMT signaling and HCC progression.

The PI3K/Akt pathway is a key signaling pathway driving survival of cancer, including HCC. Our data provide evidence that AJUBA functionally promotes EMT in HCC cells through activating the Akt/GSK-3β/Snail pathway. It is well known that p-Akt can suppress GSK-3β activity (through phosphorylation of Ser 9) and stabilize Snail, thus Snail and GSK-3β together act as a molecular switch for the PI3K/Akt pathway, regulating EMT 55. Consistent with this, we found that AJUBA overexpression increased p-Akt, p-GSK-3β, and Snail expression levels, which could be rescued by silencing AJUBA. Collectively, these data suggest that AJUBA plays an important role in EMT by activating the Akt/GSK-3β/Snail pathway. But how does AJUBA activate Akt in HCC cells? Previous reports showed that AJUBA could stabilize and enhance the association of TRAF6 with p62 by forming a protein complex 42. Moreover, TRAF6 has been found to be a direct E3 ligase for Akt and is essential for the activation of Akt through K63 ubiquitination, which is crucial for Akt membrane location and phosphorylation 40. Based on these findings, we hypothesized that the activation of Akt might be mediated by a protein complex formed by the interaction of TRAF6 and AJUBA. This was supported by the following lines of evidence: first, co-IP data provide evidence of a direct interaction between TRAF6 and AJUBA; second, there was a significant correlation between the AJUBA protein level and the TRAF6 mRNA level in HCC tissues from the TCGA database; third, AJUBA was capable of promoting the activation of Akt through enhancing TRAF6-mediated K63 ubiquitination; fourth, loss-of-function assay revealed that silencing TRAF6 could suppress cell migration and decrease the expression of mesenchymal markers (markers of EMT) induced by overexpression of AJUBA, and conversely, gain-of-function assay demonstrated that increased TRAF6 could promote cell migration and the expression of epithelial markers; finally, decreased TRAF6 reduced Akt phosphorylation, while increased TRAF6 promoted Akt phosphorylation. Our results have demonstrated an important role of AJUBA in promoting EMT via the Akt/GSK-3β/Snail pathway through its interaction with TRAF6. Further work is needed to elucidate how these components interact with each other in detail.

In summary, our study provides evidence showing that up-regulated AJUBA, mediated by TCF4 binding to AJUBA-SE in HCC, is very important in acquisition of an aggressive/poor prognostic phenotype. Furthermore, our data provide functional and mechanistic links between the SE-associated AJUBA and tumor EMT in HCC.

## Supplementary Material

Supplementary materials and methods, figures, and tables.

## Figures and Tables

**Table 1:** Correlation of AJUBA expression with patients' clinicopathological features in primary hepatocellular carcinomas

Variable	AJUBA protein			
	All cases	Low expression	High expression	*P* value^*^
**Age (years)**				0.342
≤ 50	84	64 (76.2%)	20 (23.8%)	
> 50	84	69 (82.1%)	15 (17.9%)	
**Sex**				0.519
Female	25	21 (84.0%)	4 (16.0%)	
Male	143	112 (78.3%)	31 (21.7%)	
**HbsAg**				0.321
Negative	11	10 (90.9%)	1 (9.1%)	
Positive	157	123 (78.3%)	34 (21.7%)	
**AFP (ng/mL)**				0.192
≤20	69	58 (84.1%)	11 (15.9%)	
>20	99	75 (75.8%)	24 (24.2%)	
**Liver cirrhosis**				0.885
No	99	78 (78.8%)	21 (21.2%)	
Yes	69	55 (79.7%)	14 (20.3%)	
**Tumor size (cm)**				0.443
≤5	72	59 (81.9%)	13 (18.1%)	
>5	96	74 (77.1%)	22 (22.9%)	
**Tumor multiplicity**				**0.001**
Single	131	111 (84.7%)	20 (15.3%)	
Multiple	37	22 (59.5%)	15 (40.5%)	
**Differentiation**				**0.025**
Well-moderate	86	74 (86.0%)	12 (14.0%)	
Poor-undifferentiated	82	59 (72.0%)	23 (28.0%)	
**Stage^a^**				**< 0.001**
I-II	117	102 (87.2%)	15 (12.8%)	
III-IV	51	31 (60.8%)	20 (39.2%)	
**Vascular invasion**				**0.027**
Absent	104	88 (84.6%)	16 (15.4%)	
Present	64	45 (70.3%)	19 (29.7%)	
**Relapse**				**0.003**
Absent	135	113 (83.7%)	22 (16.3%)	
Present	33	20 (60.6%)	13 (39.4%)	

Notes: a: The 8th edition of the AJCC cancer staging manual; *Chi-square test; Boldface indicates *P* < 0.05; Abbreviations: HbsAg, hepatitis B surface antigen; AFP, alpha-fetoprotein.

**Table 2:** Univariate and multivariate analysis of different prognostic features in 168 patients with hepatocellular carcinoma

Variable	Univariate analysis^*^			Multivariate analysis	
	All cases	HR (95% CI)	*P* value	HR (95% CI)	*P* value
**Age (years)**			0.462		
≤ 50	84	1.0			
> 50	84	0.779 (0.401-1.516)			
**Sex**			0.124		
Female	25	0.538 (0.244-1.185)			
Male	143	1.0			
**HbsAg**			0.385		
Positive	11	1.0			
Negative	157	2.416 (0.330-17.671)			
**AFP (ng/mL)**			0.286		
≤20	69	1.0			
>20	99	1.448 (0.733-2.862)			
**Liver cirrhosis**			0.698		
Yes	99	1.0			
No	69	1.139 (0.590-2.200)			
**Tumor size (cm)**			0.051		
≤5	72	1.0			
>5	96	2.040 (0.997-4.174)			
**Tumor multiplicity**			**0.001**		0.719
Single	131	1.0		1.0	
Multiple	37	3.095 (1.591-6.022)		1.164 (0.509-2.659)	
**Differentiation**			**0.005**		0.141
Well-moderate	86	1.0		1.0	
Poor-undifferentiated	82	2.749 (1.350-5.598)		1.749 (0.831-3.680)	
**Stage^a^**			**< 0.001**		**0.032**
I-II	117	1.0		1.0	
III-IV	51	4.357 (2.227-8.527)		2.580 (1.084-6.140)	
**Vascular invasion**			0.096		
Absent	104	1.0			
Present	64	1.745 (0.906-3.361)			
**Relapse**			0.886		
Absent	135	1.0			
Present	33	1.060 (0.481-2.336)			
**AJUBA expression**			**< 0.001**		**0.035**
Low	133	1.0		1.0	
High	35	3.950 (2.052-7.602)		2.189 (1.058-4.529)	

Notes: a: The 8th edition of the AJCC cancer staging manual; *Cox regression model; Boldface indicates *P* < 0.05; Abbreviations: HbsAg, hepatitis B surface antigen; AFP, alpha-fetoprotein; HR indicates hazards ratio; CI indicates confidence interval.

## Abbreviations

AJUBA: ajuba LIM protein
BiFC: bimolecular fluorescence complementation
co-IP: co-Immunoprecipitation
ChIP-seq: chromatin immunoprecipitation sequencing
CCLE: Cancer Cell Line Encyclopedia project
CA-AKT: constitutively active Akt gene
EMT: epithelial-mesenchymal transition
FBS: fetal bovine serum
HCC: hepatocellular carcinoma
H&E: haematoxylin and eosin
HiC: high throughput chromosome conformation capture
H3K27ac: H3K27 acetylation
H3K4me1: monomethylation of the 4th lysine of histone 3
H3K4me3: trimethylation of the 4th lysine of histone 3
IHC: immunohistochemistry
IF: immunofluorescence
IGV: integrative genomics viewer
ICE: iterative correction and eigenvector decomposition
IP: immunoprecipitation
KEGG: Kyoto Encyclopedia of Genes and Genomes
NC: negative control
PBS: phosphate buffered solution
Pol 2: RNA polymerase 2
p-Akt: phosphorylated Akt
p-GSK-3β: phosphorylated GSK-3β
ROSE: rank ordering of super enhancers
SD: standard deviation
SEs: super-enhancers
TCF4: transcription factor 4
TCGA: The Cancer Genome Atlas
TMA: tissue microarray
TNM: Tumor, node, metastasis
TRAF6: tumor necrosis factor associated factor 6

## References

[1] Bupathi M, Kaseb A, Meric-Bernstam F, Naing A (2015). Hepatocellular carcinoma: Where there is unmet need. Mol Oncol.

[2] Kim WR, Gores GJ (2013). Recurrent hepatocellular carcinoma: it's the virus!. J Clin Oncol.

[3] Moreno-Bueno G, Portillo F, Cano A (2008). Transcriptional regulation of cell polarity in EMT and cancer. Oncogene.

[4] Thiery JP, Acloque H, Huang RY, Nieto MA (2009). Epithelial-mesenchymal transitions in development and disease. Cell.

[5] Lamouille S, Xu J, Derynck R (2014). Molecular mechanisms of epithelial-mesenchymal transition. Nat Rev Mol Cell Biol.

[6] Yang MH, Chen CL, Chau GY, Chiou SH, Su CW, Chou TY, Peng WL, Wu JC (2009). Comprehensive analysis of the independent effect of twist and snail in promoting metastasis of hepatocellular carcinoma. Hepatology.

[7] Peng L, Jiang B, Yuan X, Qiu Y, Peng J, Huang Y, Zhang C, Zhang Y, Lin Z, Li J (2019). Super-Enhancer-Associated Long Noncoding RNA HCCL5 Is Activated by ZEB1 and Promotes the Malignancy of Hepatocellular Carcinoma. Cancer Res.

[8] Jia Y, Chng WJ, Zhou J (2019). Super-enhancers: critical roles and therapeutic targets in hematologic malignancies. J Hematol Oncol.

[9] Hnisz D, Abraham BJ, Lee TI, Lau A, Saint-Andre V, Sigova AA, Hoke HA, Young RA (2013). Super-enhancers in the control of cell identity and disease. Cell.

[10] Lin DC, Dinh HQ, Xie JJ, Mayakonda A, Silva TC, Jiang YY, Ding LW, He JZ, Xu XE, Hao JJ (2018). Identification of distinct mutational patterns and new driver genes in oesophageal squamous cell carcinomas and adenocarcinomas. Gut.

[11] Riggi N, Knoechel B, Gillespie SM, Rheinbay E, Boulay G, Suva ML, Rossetti NE, Boonseng WE, Oksuz O, Cook EB (2014). EWS-FLI1 utilizes divergent chromatin remodeling mechanisms to directly activate or repress enhancer elements in Ewing sarcoma. Cancer Cell.

[12] Jiang YY, Lin DC, Mayakonda A, Hazawa M, Ding LW, Chien WW, Xu L, Chen Y, Xiao JF, Senapedis W (2017). Targeting super-enhancer-associated oncogenes in oesophageal squamous cell carcinoma. Gut.

[13] Yuan J, Jiang YY, Mayakonda A, Huang M, Ding LW, Lin H, Yu F, Lu Y, Loh TKS, Chow M (2017). Super-Enhancers Promote Transcriptional Dysregulation in Nasopharyngeal Carcinoma. Cancer Res.

[14] Eliades P, Abraham BJ, Ji Z, Miller DM, Christensen CL, Kwiatkowski N, Kumar R, Njauw CN, Taylor M, Miao B (2018). High MITF Expression Is Associated with Super-Enhancers and Suppressed by CDK7 Inhibition in Melanoma. J Invest Dermatol.

[15] van Groningen T, Koster J, Valentijn LJ, Zwijnenburg DA, Akogul N, Hasselt NE, Broekmans M, Haneveld F, Nowakowska NE, Bras J (2017). Neuroblastoma is composed of two super-enhancer-associated differentiation states. Nat Genet.

[16] Kanungo J, Pratt SJ, Marie H, Longmore GD (2000). Ajuba, a cytosolic LIM protein, shuttles into the nucleus and affects embryonal cell proliferation and fate decisions. Mol Biol Cell.

[17] Kalan S, Matveyenko A, Loayza D (2013). LIM Protein Ajuba Participates in the Repression of the ATR-Mediated DNA Damage Response. Front Genet.

[18] Reddy BV, Irvine KD (2013). Regulation of Hippo signaling by EGFR-MAPK signaling through Ajuba family proteins. Dev Cell.

[19] Zhu W, Cai MY, Tong ZT, Dong SS, Mai SJ, Liao YJ, Bian XW, Lin MC, Kung HF, Zeng YX (2012). Overexpression of EIF5A2 promotes colorectal carcinoma cell aggressiveness by upregulating MTA1 through C-myc to induce epithelial-mesenchymaltransition. Gut.

[20] Cai MY, Zhang B, He WP, Yang GF, Rao HL, Rao ZY, Wu QL, Guan XY, Kung HF, Zeng YX (2010). Decreased expression of PinX1 protein is correlated with tumor development and is a new independent poor prognostic factor in ovarian carcinoma. Cancer Sci.

[21] Camp RL, Dolled-Filhart M, Rimm DL (2004). X-tile: a new bio-informatics tool for biomarker assessment and outcome-based cut-point optimization. Clin Cancer Res.

[22] Servant N, Varoquaux N, Lajoie BR, Viara E, Chen CJ, Vert JP, Heard E, Dekker J, Barillot E (2015). HiC-Pro: an optimized and flexible pipeline for Hi-C data processing. Genome Biol.

[23] Loven J, Hoke HA, Lin CY, Lau A, Orlando DA, Vakoc CR, Bradner JE, Lee TI, Young RA (2013). Selective inhibition of tumor oncogenes by disruption of super-enhancers. Cell.

[24] Zhang Z, Ma P, Jing Y, Yan Y, Cai MC, Zhang M, Zhang S, Peng H, Ji ZL, Di W (2016). BET Bromodomain Inhibition as a Therapeutic Strategy in Ovarian Cancer by Downregulating FoxM1. Theranostics.

[25] Puissant A, Frumm SM, Alexe G, Bassil CF, Qi J, Chanthery YH, Nekritz EA, Zeid R, Gustafson WC, Greninger P (2013). Targeting MYCN in neuroblastoma by BET bromodomain inhibition. Cancer Discov.

[26] Chapuy B, McKeown MR, Lin CY, Monti S, Roemer MG, Qi J, Rahl PB, Sun HH, Yeda KT, Doench JG (2013). Discovery and characterization of super-enhancer-associated dependencies in diffuse large B cell lymphoma. Cancer Cell.

[27] Lin L, Huang M, Shi X, Mayakonda A, Hu K, Jiang YY, Guo X, Chen L, Pang B, Doan N (2019). Super-enhancer-associated MEIS1 promotes transcriptional dysregulation in Ewing sarcoma in co-operation with EWS-FLI1. Nucleic Acids Res.

[28] Dixon JR, Selvaraj S, Yue F, Kim A, Li Y, Shen Y, Hu M, Liu JS, Ren B (2012). Topological domains in mammalian genomes identified by analysis of chromatin interactions. Nature.

[29] Comprehensive and Integrative Genomic Characterization of Hepatocellular Carcinoma. Cell 2017;169:1327-1341.e1323 10.1016/j.cell.2017.05.046 PMC5680778 28622513

[30] Whyte WA, Orlando DA, Hnisz D, Abraham BJ, Lin CY, Kagey MH, Rahl PB, Lee TI, Young RA (2013). Master transcription factors and mediator establish super-enhancers at key cell identity genes. Cell.

[31] Parker SC, Stitzel ML, Taylor DL, Orozco JM, Erdos MR, Akiyama JA, van Bueren KL, Chines PS, Narisu N, Black BL (2013). Chromatin stretch enhancer states drive cell-specific gene regulation and harbor human disease risk variants. Proc Natl Acad Sci U S A.

[32] Pastushenko I, Blanpain C (2019). EMT Transition States during Tumor Progression and Metastasis. Trends Cell Biol.

[33] David CJ, Huang YH, Chen M, Su J, Zou Y, Bardeesy N, Iacobuzio-Donahue CA, Massague J (2016). TGF-beta Tumor Suppression through a Lethal EMT. Cell.

[34] Niederst MJ, Benes CH (2014). EMT twists the road to PI3K. Cancer Discov.

[35] Qi J, Yu Y, Akilli Ozturk O, Holland JD, Besser D, Fritzmann J, Wulf-Goldenberg A, Eckert K, Fichtner I, Birchmeier W (2016). New Wnt/beta-catenin target genes promote experimental metastasis and migration of colorectal cancer cells through different signals. Gut.

[36] Zheng T, Yin D, Lu Z, Wang J, Li Y, Chen X, Liang Y, Song X, Qi S, Sun B (2014). Nutlin-3 overcomes arsenic trioxide resistance and tumor metastasis mediated by mutant p53 in Hepatocellular Carcinoma. Mol Cancer.

[37] Puisieux A, Brabletz T, Caramel J (2014). Oncogenic roles of EMT-inducing transcription factors. Nat Cell Biol.

[38] Kudo-Saito C, Shirako H, Takeuchi T, Kawakami Y (2009). Cancer metastasis is accelerated through immunosuppression during Snail-induced EMT of cancer cells. Cancer Cell.

[39] Liu L, Dai Y, Chen J, Zeng T, Li Y, Chen L, Zhu YH, Li J, Li Y, Ma S (2014). Maelstrom promotes hepatocellular carcinoma metastasis by inducing epithelial-mesenchymal transition by way of Akt/GSK-3beta/Snail signaling. Hepatology.

[40] Yang WL, Wang J, Chan CH, Lee SW, Campos AD, Lamothe B, Hur L, Grabiner BC, Lin X, Darnay BG (2009). The E3 ligase TRAF6 regulates Akt ubiquitination and activation. Science.

[41] Zhang L, Wang Y, Xiao F, Wang S, Xing G, Li Y, Yin X, Lu K, Wei R, Fan J (2014). CKIP-1 regulates macrophage proliferation by inhibiting TRAF6-mediated Akt activation. Cell Res.

[42] Feng Y, Longmore GD (2005). The LIM protein Ajuba influences interleukin-1-induced NF-kappaB activation by affecting the assembly and activity of the protein kinase Czeta/p62/TRAF6 signaling complex. Mol Cell Biol.

[43] Yang L, Qiu J, Xiao Y, Hu X, Liu Q, Chen L, Huang W, Li X, Li L, Zhang J (2018). AP-2β inhibits hepatocellular carcinoma invasion and metastasis through Slug and Snail to suppress epithelial-mesenchymal transition. Theranostics.

[44] Kang Y, Massague J (2004). Epithelial-mesenchymal transitions: twist in development and metastasis. Cell.

[45] Giannelli G, Koudelkova P, Dituri F, Mikulits W (2016). Role of epithelial to mesenchymal transition in hepatocellular carcinoma. J Hepatol.

[46] Fang JH, Zhou HC, Zhang C, Shang LR, Zhang L, Xu J, Zheng L, Yuan Y, Guo RP, Jia WH (2015). A novel vascular pattern promotes metastasis of hepatocellular carcinoma in an epithelial-mesenchymal transition-independent manner. Hepatology.

[47] Xu Q, Liu X, Liu Z, Zhou Z, Wang Y, Tu J, Li L, Bao H, Yang L, Tu K (2017). MicroRNA-1296 inhibits metastasis and epithelial-mesenchymal transition of hepatocellular carcinoma by targeting SRPK1-mediated PI3K/AKT pathway. Mol Cancer.

[48] Hou Z, Peng H, Ayyanathan K, Yan KP, Langer EM, Longmore GD, Rauscher FJ 3rd (2008). The LIM protein AJUBA recruits protein arginine methyltransferase 5 to mediate SNAIL-dependent transcriptional repression. Mol Cell Biol.

[49] Jiang Y, Jiang YY, Xie JJ, Mayakonda A, Hazawa M, Chen L, Xiao JF, Li CQ, Huang ML, Ding LW (2018). Co-activation of super-enhancer-driven CCAT1 by TP63 and SOX2 promotes squamous cancer progression. Nat Commun.

[50] Xie JJ, Jiang YY, Jiang Y, Li CQ, Lim MC, An O, Mayakonda A, Ding LW, Long L, Sun C (2018). Super-Enhancer-Driven Long Non-Coding RNA LINC01503, Regulated by TP63, Is Over-Expressed and Oncogenic in Squamous Cell Carcinoma. Gastroenterology.

[51] Taniguchi K, Roberts LR, Aderca IN, Dong X, Qian C, Murphy LM, Nagorney DM, Burgart LJ, Roche PC, Smith DI (2002). Mutational spectrum of beta-catenin, AXIN1, and AXIN2 in hepatocellular carcinomas and hepatoblastomas. Oncogene.

[52] Hovanes K, Li TW, Munguia JE, Truong T, Milovanovic T, Lawrence Marsh J, Holcombe RF, Waterman ML (2001). Beta-catenin-sensitive isoforms of lymphoid enhancer factor-1 are selectively expressed in colon cancer. Nat Genet.

[53] Zhao DH, Hong JJ, Guo SY, Yang RL, Yuan J, Wen CY, Zhou KY, Li CJ (2004). Aberrant expression and function of TCF4 in the proliferation of hepatocellular carcinoma cell line BEL-7402. Cell Res.

[54] Han J, Meng J, Chen S, Wang X, Yin S, Zhang Q, Liu H, Qin R, Li Z, Zhong W (2019). YY1 Complex Promotes Quaking Expression via Super-Enhancer Binding during EMT of Hepatocellular Carcinoma. Cancer Res.

[55] Zhou BP, Deng J, Xia W, Xu J, Li YM, Gunduz M, Hung MC (2004). Dual regulation of Snail by GSK-3beta-mediated phosphorylation in control of epithelial-mesenchymal transition. Nat Cell Biol.

