# Supplementary material for: Super-enhancer-driven AJUBA is activated by TCF4 and involved in epithelial-mesenchymal transition in the progression of Hepatocellular Carcinoma
Source: Theranostics. 2020 Jul 11;10(20):9066–82. doi: 10.7150/thno.45349 (PMC7415796; doi:10.7150/thno.45349)
Supplement: Supplementary file 1 — Supplementary materials and methods, figures, and tables. [file thnov10p9066s1.pdf]

Super-enhancer-driven AJUBA is activated by TCF4 and involved in epithelial-mesenchymal transition in the progression of hepatocellular carcinoma

Chi Zhang[1,#], Shi Wei [1,#], Wei-Peng Sun[2,#], Kai Teng[3], Miao-Miao Dai[1], Feng-Wei Wang[1], Jie-Wei Chen[4], Han Ling[1], Xiao-Dan Ma[1], Zi-Hao Feng[5], Jin-Ling Duan[1], Mu-Yan Cai[4,*], Dan Xie[1,4,*]

[1]State Key Laboratory of Oncology in South China, Collaborative Innovation Center for Cancer Medicine, Sun Yat-sen University Cancer Center, 510060, Guangzhou, China, [2]Department of Anorectal Surgery, The First Affiliated Hospital of Zhengzhou University, 450052, Zhengzhou, China, [3]Department of Radiation Oncology, Shandong Cancer Hospital and Institute, Shandong First Medical University and Shandong Academy of Medical Sciences, 271000, Jinan, China, [4]Department of Pathology, Sun Yat-sen University Cancer Center, 510060, Guangzhou, China, [5]Department of Urology, First Affiliated Hospital, Sun Yat-sen University, 510060, Guangzhou, China.

[#]These authors contributed equally to this work.

[*]Correspondence: xiedan@sysucc.org.cn and caimy@sysucc.org.cn

Email addresses for all authors: Chi Zhang (zhangchi@sysucc.org.cn)

Shi Wei (weishi@sysucc.org.cn)

Wei-Peng Sun (levinsun528@163.com)

Kai Teng (tengkaitk@126.com)

Miao-Miao Dai (daimm@sysucc.org.cn)

Feng-Wei Wang (wangfengw@sysucc.org.cn)

Jie-Wei Chen (chenjiew@sysucc.org.cn)

Han Ling (linghan@sysucc.org.cn)

Xiao-Dan Ma (maxd@sysucc.org.cn)

Zi-Hao Feng: zerofung1991@163.com

Jin-Ling Duan: duanjl@sysucc.org.cn

Mu-Yan Cai: caimy@sysucc.org.cn

Dan Xie: xiedan@sysucc.org.cn

Supplemental materials and methods

Western blot and antibodies

Western blotting was performed in accordance with the standard methods. Briefly, cells were lysed in RIPA buffer (Thermo-Fisher Scientific, Waltham, MA, USA) containing proteinase inhibitor cocktail (Roche, Basel, Switzerland) for 30 min on ice. Then, protein concentration was measured using a BCA kit (Thermo-Fisher Scientific, Waltham, MA, USA). Western blotting was performed using SDS-PAGE followed by transfer to a 0.22 μm PVDF membrane (Roche, Basel, Switzerland). The membrane was incubated with primary antibody overnight at 4 °C followed by incubation with secondary antibody for 1–2 h at room temperature. Immunoreactivity was visualized using an ECL chemiluminescence system (Tanon, Shanghai, China). Primary antibodies used were: AJUBA (HPA006171, Sigma-Aldrich, St. Louis, MO, USA), E-cadherin (610182, BD Transduction Laboratories, Franklin Lakes, New Jersey, Biosciences, USA), N-cadherin (610921, BD Transduction Laboratories, Franklin Lakes, New Jersey, USA), vimentin (550513, BD Transduction Laboratories, Franklin Lakes, New Jersey, USA), β-catenin (6101054, BD Transduction Laboratories, Franklin Lakes, New Jersey, USA), Akt (#4691, Cell Signaling Technology, CA, USA), p-Akt (#4060, Cell Signaling Technology, CA, USA), GSK-3β (#12456, Cell Signaling Technology, CA, USA), p-GSK-3β (#5558, Cell Signaling Technology, CA, USA), Snail (#3879, Cell Signaling Technology, CA, USA), c-myc (#5605, Cell Signaling Technology, CA, USA), cyclinD1 (#2978, Cell Signaling Technology, CA, USA), TCF4 (#2565, Cell Signaling Technology, CA, USA), MET (A0040, ABclonal, Wuhan, China), TRAF6 (ab40675, Abcam, Cambridge, MA, USA), Flag (M20018, Abmart, Shanghai, China), Myc (M047-3, MBL, Japan), and GAPDH (60004, Proteintech Group Inc, USA). All western blot bands were quantified using Image J software.

RNA extraction and real-time PCR

Total RNA was extracted from tissues or cell culture samples using the TRIzol reagent (Invitrogen, Carlsbad, CA, USA) according to the manufacturer's protocol, and cDNA was obtained from the total RNA using a PrimeScript RT-PCR kit (Takara, Tokyo, Japan). Target mRNA levels were determined by performing qRT-PCR with a SYBR® Green PCR kit (Bio-Rad, USA) in the LightCycler® 480II (Roche, Basel, Switzerland). GAPDH expression served as the internal control. The primer sequences are shown below (Table S1).

Table S1 Primer sequences used for quantitative RT-PCR

Gene	Primer Sequence (5′ to 3′)	Product size (bp)
AJUBA	F: GATGCGGGAGCCAGAGG<br>R: CACAAGAGCAGCAAACAAAGC	137
E-cadherin	F: CGAGAGCTACACGTTCACGG<br>R: GGGTGTCGAGGGAAAAATAGG	119
N-cadherin	F: TTTGATGGAGGTCTCCTAACACC	120

	R: ACGTTTAACACGTTGGAAATGTG	
β-catenin	F: AAAGCGGCTGTTAGTCACTGG<br>R: CGAGTCATTGCATACTGTCCAT	215
Vimentin	F: GAGAACTTTGCCGTTGAAGC<br>R: GCTTCCTGTAGGTGGCAATC	163
TCF4	F: AGAAACGAATCAAAACAGCTCCT<br>R: CGGGATTTGTCTCGGAAACTT	84
TRAF6	F: TTTGCTCTTATGGATTGTCCCC<br>R: CATTGATGCAGCACAGTTGTC	120
GAPDH	F: TGCACCACCAACTGCTTAGC<br>R: GGCATGGACTGTGGTCATGAG	87
ChIP-E1A	F: AGGGGAAGTTGGCTGGAATG<br>R: AACTGCTGTCTGTCCTTCCC	84
ChIP-E1B	F: GAGCAGTGACCCCAGAAGTC<br>R: AACGCAAAGTTCGCCCTAGA	85
ChIP-E2A	F: GGTCAGAGTTCAGCCACCTC<br>R: GCATTCCTGGAGCGTGATGT	77
ChIP-E2B	F: GGAGGCCCACTTGGAAAGG<br>R: TGGGGGAGTGAGAAAAGCTG	166
ChIP-E4A	F: GGTATTCGGTTCTCCACTGCC<br>R: TCCTAGTCCCGCGTCTTCAC	77
ChIP-E4B	F: TCTCGTGAGTCCGTTTCTCA<br>R: CACTTGGTCCCTTCCCTACG	76

Plasmids, small interfering RNAs, and transfection

Full-length AJUBA and TRAF6 complementary DNA were amplified via RT-PCR. Then, AJUBA was cloned into the pLVX-myc-3xflag lentivirus vector and TRAF6 was ligated to the MC-Flag-pCS2 plasmid. The MC-Flag-pcDNA3.1-CA-AKT plasmid was purchased from Wuhan GeneCreate Co, Ltd. Short hairpin RNA (shRNA) targeted to human AJUBA or the corresponding control vectors (psi-LVRU6GP) were designed and purchased from GeneCopoeia (Rockville, USA). All lentiviral particles were prepared in HEK293T cells. For lentiviral particle production, recombinant vectors were co-transfected with packaging vectors (psPAX2 and pMD2.G) using Lipofectamine 2000 (Invitrogen, Carlsbad, CA, USA). At 48 h post transfection, supernatants containing viral particles were harvested and filtered (pore size: 0.45 μm) and stored at -80 °C. To generate stable cell lines, HCC cells were transduced with virus-containing medium in the presence of 8 μg/mL polybrene for 16 h, and selected in the presence of 2 μg/mL puromycin (2 μg/mL, Invitrogen, Carlsbad, CA, USA) for stably transduced cells. Small interfering RNAs (siRNAs) against TCF4 and TRAF6 were obtained from RiboBio Company (Guangzhou, China). Lipofectamine™ RNAiMAX (Invitrogen, Carlsbad, CA, USA) was used for transfection in

accordance with the manufacturer's instructions. The cloning primers, shRNA sequences, and siRNA sequences are listed below (Table S2).

Table S2 Cloning primer, shRNA, and siRNA sequences

Gene	Primer Sequence (5′ to 3′)
AJUBA	F: AATACGCGTATGGAGCGGTTAGGAGAAAG
	R: AATGGATCCTCAGATATAGTTGGCAGGGGG
TRAF6	F: AATGAATTCATGAGTCTGCTAAACTGTGAA
	R: AATCTCGAGTACCCCTGCATCAGTACTTCG
AJUBA-shNC	ACAGAAGCGATTGTTGATC
AJUBA-sh35	GCATGAGTCACGCAATGTTCT
AJUBA-sh36	GGAGTAGAGGACCTAGCTTTA
TCF4-si01	GGATTTAGCTGATGTCAAA
TCF4-si03	CAACCAGTGTACCCAATCA
TRAF6-si01	GTAGCGCTGTAACAAAAGA
TRAF6-si02	AGGGTCGCCTTGTAAGACA

Wound healing assay

Briefly, HCC cells were seeded into 6-well plates, and when cells reached approximately 90% confluence, the cell monolayer was scratched with a sterile 10 μL pipette tip and then washed three times with PBS. Cells were then incubated in DMEM containing 1% FBS for 48 h. At both 0 h and 48 h, cell scratches were photographed under an inverted microscope (200×, Nikon, Japan), and the gap size was measured and calculated using Image J. All experiments were performed independently in triplicate.

CRISPR-Cas9 genomic editing

The CRISPR-Cas9 system was used to delete E2 in HepG2 cells according to the following procedure. Specific guide RNAs (sgRNA) were designed by using a web tool (http: //crispr.mit.edu/). The sgRNAs were then cloned into Cas9 expressing pSpCas9(BB)-2A-Puro (PX459) V2.0 plasmid (Addgene, Cat# 62988) using BsbI site. HepG2 cells were transfected with two constructed plasmids using Lipofectamine 3000 (Invitrogen, Carlsbad, CA, USA). 48 h after transfection, 1 μg/mL puromycin treated for several days until all the cells died in the control plate. For clone isolation, cells were sorted into 96-well plates to obtain single cell clones. Individual colonies were grown for approximately 4 weeks prior to DNA extraction for PCR validation and DNA sequencing. All the sgRNAs and genotyping PCR primers are listed below (Table S3).

Table S3 sgRNAs and genotyping PCR primers sequences

	Sequence (5′ to 3′)
sg1	F: CACCGCACGTATTCCAGCACGTAGT
	R: AAACACTACGTGCTGGAATACGTGC
sg2	F: CACCGCGGACCTCCAGACACTTGG
	R: AAACCCAAGTGTCTGGAGGTCCGC
P1	CCTTCCCCGCACAAAAATTC
P2	CCCCTGCCCACATACACACT
P3	AGAACAGGTGGAGCATGAGC
P4	GGTCAGAGTTCAGCCACCTC

Supplementary data

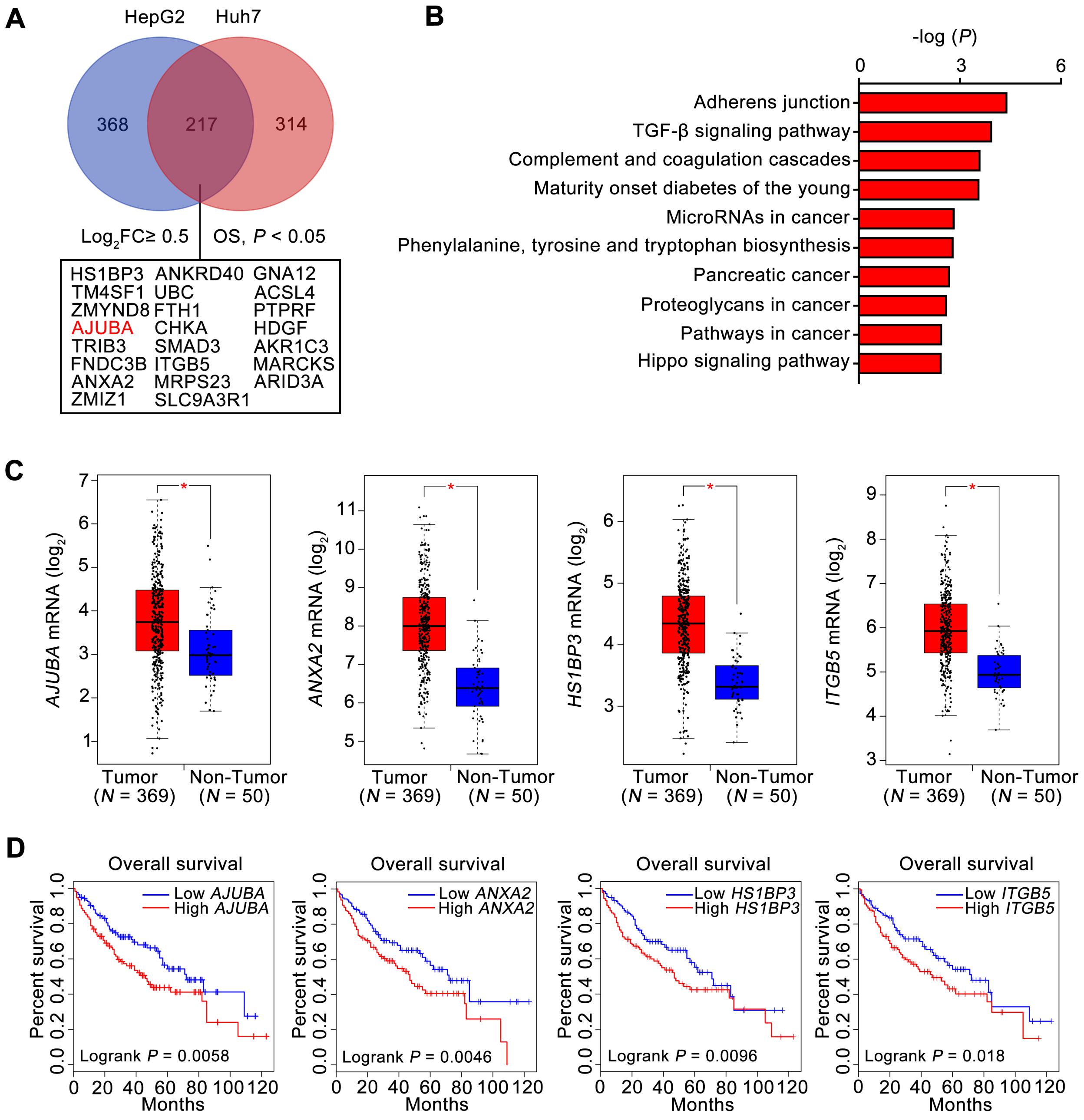

Figure S1. Identification of potential super-enhancer associated genes related to EMT. (A) Venn diagram shows the super-enhancer target genes overlapping between HepG2 and Huh7 cells. HepG2 and Huh7 cell data were re-analyzed based on the ENCODE project. The gene list shown below was selected based on the logarithm of T-N fold change to the base 2 ($log_2FC \geq 0.5$) and overall survival ($P < 0.05$). (B) Major functional pathways modulated by the overlapping super-enhancer target genes based on KEGG pathway analysis. (C) Scatter plots showing relative levels of multiple target gene mRNAs in non-tumor and tumor tissues. Median expression levels in each group are indicated by horizontal lines. One-way ANOVA; $^*P < 0.05$. (D) Overall survival curves for HCC patients with high and low target gene expression levels. The data were obtained from TCGA datasets. Significance of the differences between the two categories was determined by Log-rank test.

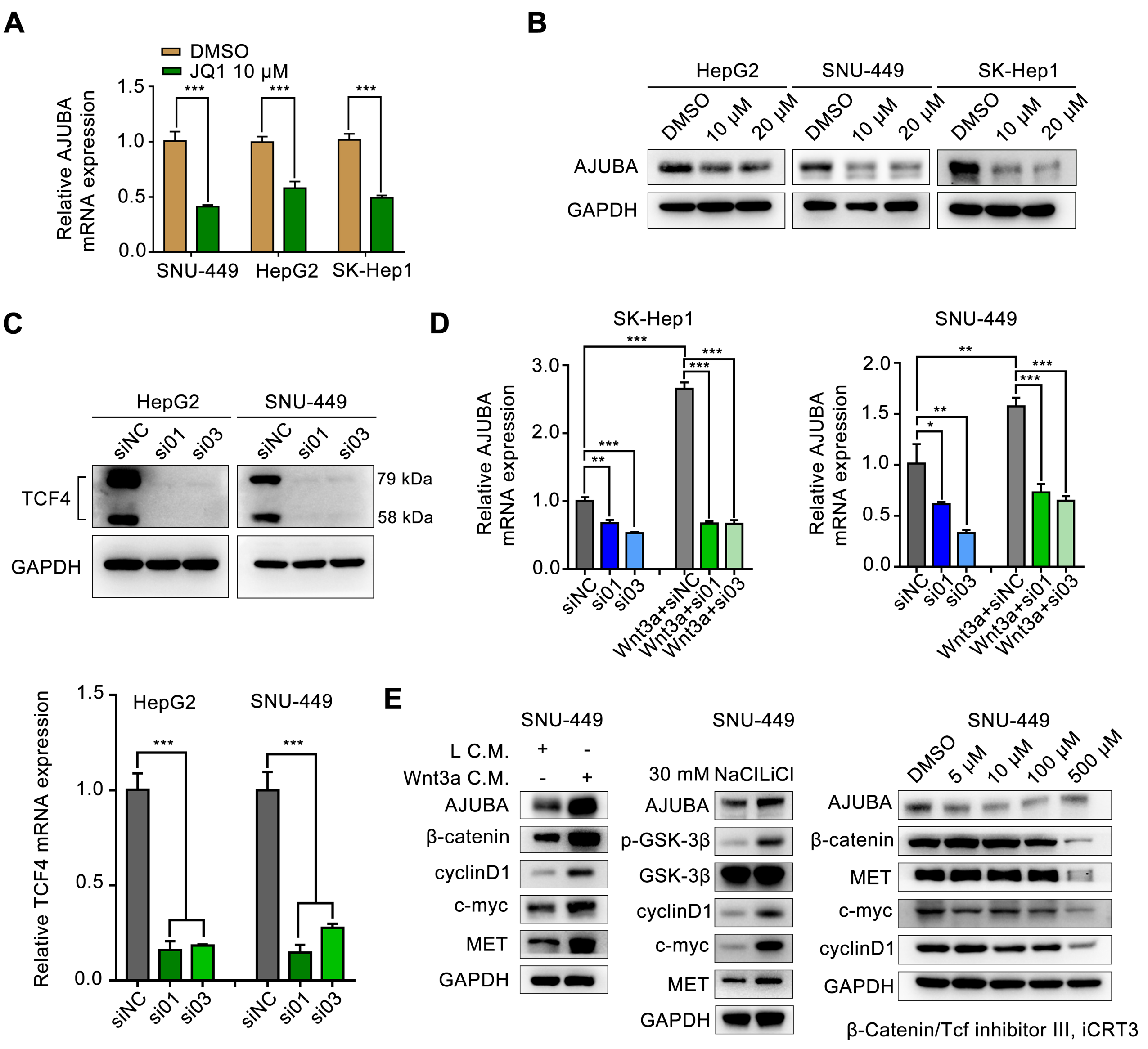

Figure S2. AJUBA expression was regulated by its super-enhancer and TCF4 in HCC cells. AJUBA qRT-PCR (A) and western blot (B) analysis of HCC cell lines treated with DMSO or JQ1 for 24 h. (C) The knockdown efficiency of TCF4 was measured using qRT-PCR and western blotting. (D) The relative expression levels of AJUBA mRNA after silencing TCF4 in SK-Hep1 and SNU-449 cell lines with or without Wnt3a C.M. stimulation overnight. Data are represented as mean ± SD and relative to DMSO. mRNA levels are normalized to GAPDH. *$P < 0.05$, **$P < 0.01$, ***$P < 0.001$. (E) The expression of AJUBA and Wnt pathway components in SNU-449 cells treated with either L C.M. or Wnt3a C.M. for 24 h. (left panel). The protein levels of AJUBA and Wnt pathway components in SNU-449 cells treated with LiCl (middle panel) or iCRT3 (right panel). Abbreviations: DMSO, dimethyl sulfoxide.

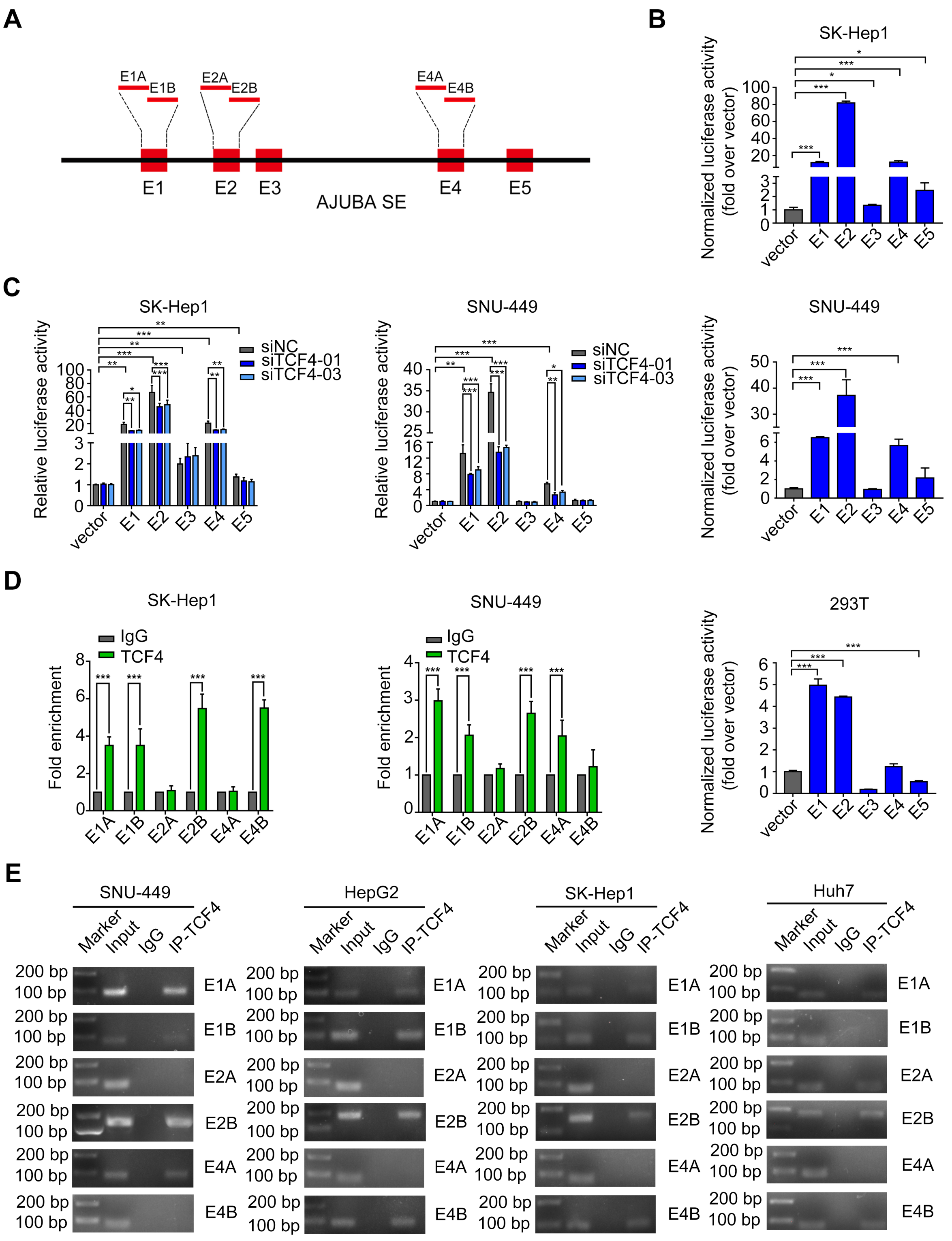

Figure S3. TCF4 directly binds to super-enhancer regions of AJUBA. (A) Constituent enhancers (E1, E2, and E4) were further divided into two constituents (E1A, E1B, E2A, E2B, E4A, and E4B) for ChIP-qPCR. (B) Relative luciferase activity upon transfection of each enhancer (E1-E5) in SK-Hep1, SNU-449 and HEK293T cells. (C) The luciferase activities of each of the five enhancer elements measured in SK-Hep1 and SNU-449 cells after silencing TCF4. For (B and C), luciferase signal was normalized to a renilla transfection control. (D) ChIP-qPCR analysis of the interaction between TCF4 and segments of E1, E2, and E4 (E1A, E1B, E2A, E2B, E4A, and E4B) in SK-Hep1 and SNU-449 cells. Rabbit normal IgG antibody was used as a negative control. Data in B-D are representative of 2-3 separate experiments. (E) DNA gel analysis of the relative fold enrichment of the E1, E2, and E4 fragments with TCF4 or rabbit normal IgG antibodies in ChIP experiments. Three independent experiments were performed. All error bars show standard error of the mean. *$P < 0.05$, **$P < 0.01$, ***$P < 0.001$. Abbreviations: SE, super-enhancer.

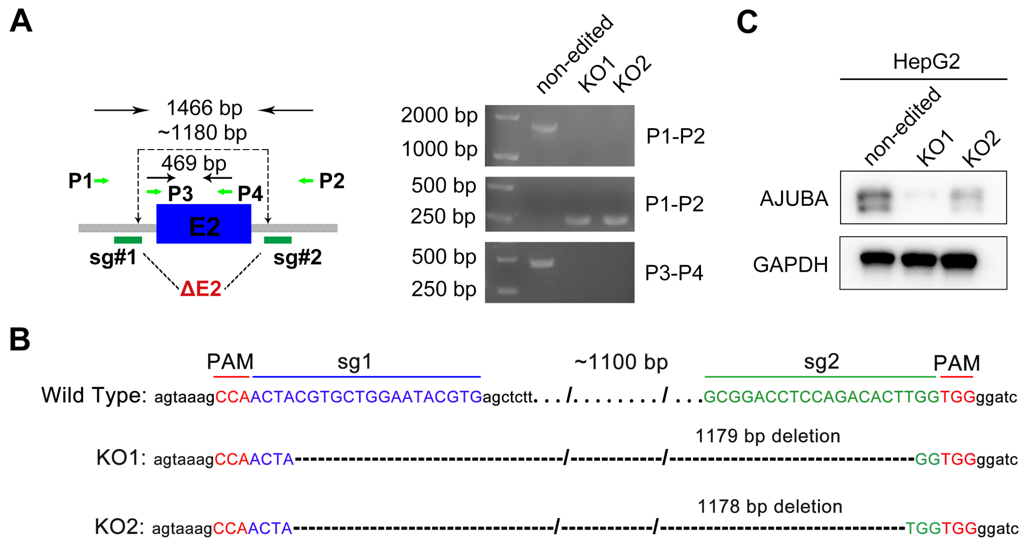

Figure S4. AJUBA expression is regulated by its putative SE. (A) Schematic of E2 element showing the sgRNAs (sg1 and sg2) used to delete E2 element, as well as the primers (P1-P4) used to screen the CRISPR-Cas9-edited cells by PCR (left). Agarose gel electrophoresis results showing deletions as indicated (right). Non-edited clone was shown as negative control. (B) DNA sequences of the edited region for KO clones. The sgRNA target sites and PAM regions are highlighted. (C) CRISPR-Cas9-mediated editing of constituent enhancer E2. AJUBA protein levels were measured in edited HepG2 cells. Non-edited clone was used as negative control. GAPDH was used as a loading control.

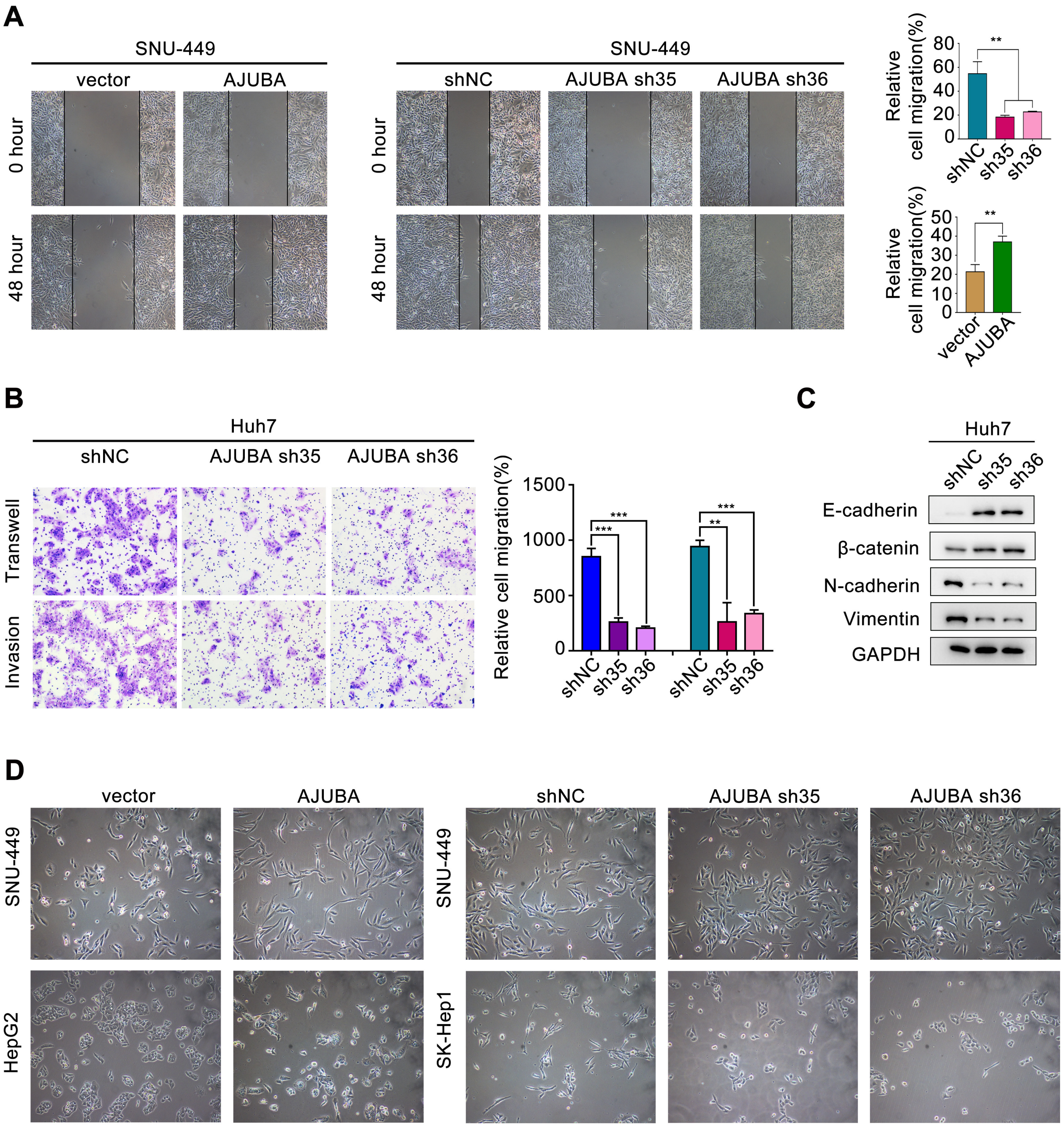

Figure S5. AJUBA promotes HCC migration and EMT *in vitro*. (A) Wound healing assays were performed to assess cell motility in AJUBA ectopic expression and depletion SNU-449 cell lines by measuring a scratch after 48 h. (B) Transwell migration and matrigel invasion assays were performed in Huh7 cells with AJUBA knockdown. The right panel shows bar charts quantifying cell migration and invasion. Three independent experiments were performed, and quantitative data are presented as mean ± SD. $^{**}P < 0.01$, $^{***}P < 0.001$. (C) The protein levels of EMT markers (E-cadherin, β-catenin, N-cadherin, and vimentin) in Huh7 cells with AJUBA knockdown. (D) Cell morphology of cells with AJUBA-knockdown or -overexpression. Abbreviations: shNC, negative control short hairpin RNA; sh35, NO.35 short hairpin RNA for AJUBA; sh36, NO.36 short hairpin RNA for AJUBA.

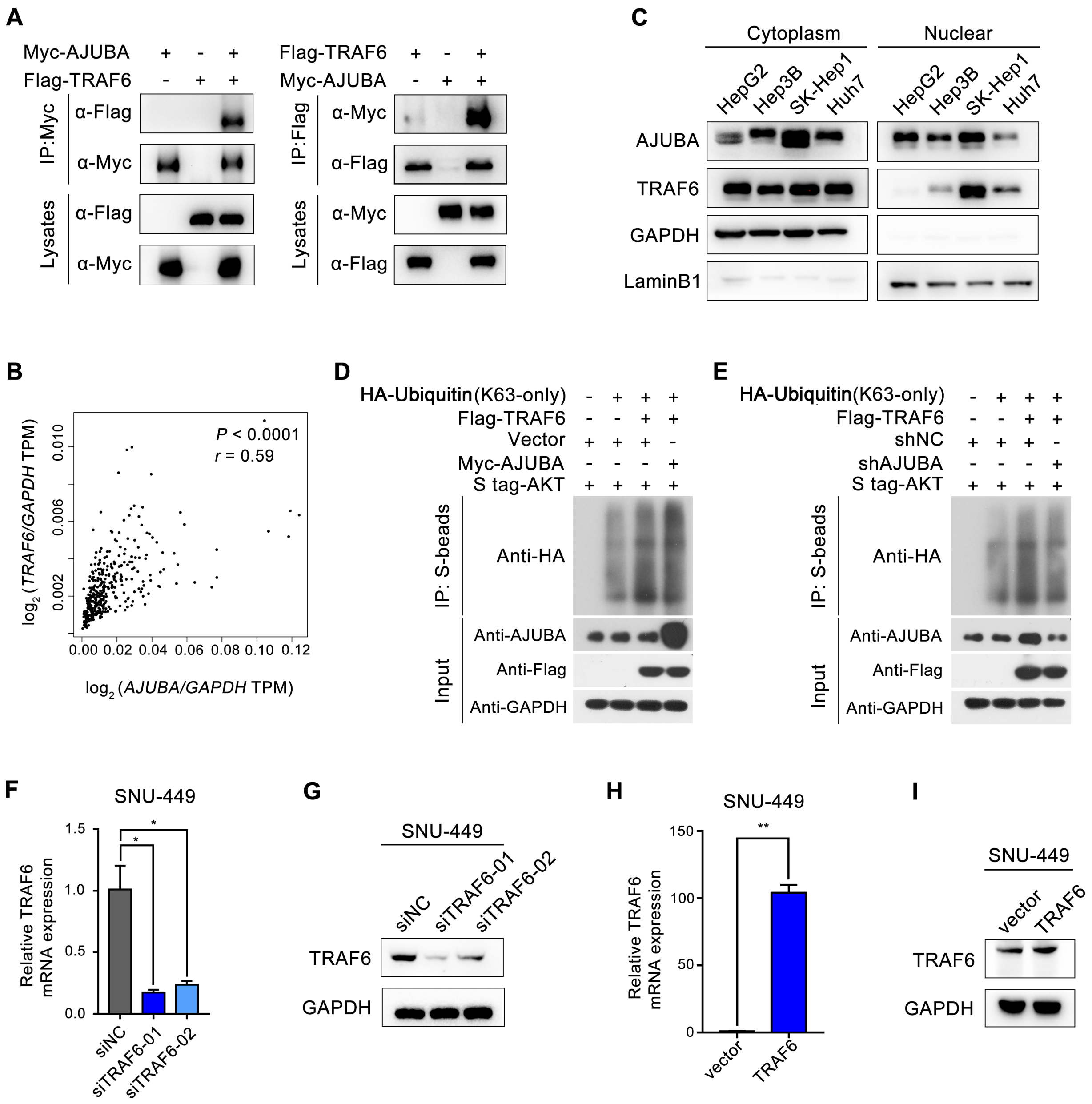

Figure S6. AJUBA interacts with TRAF6. (A) Flag-tagged TRAF6 interacted with Myc-tagged AJUBA by co-IP in HEK293T cells with indicated antibodies. (B) The correlation of TRAF6 and AJUBA mRNA levels, normalized by GAPDH, in HCC tissues analyzed using the GEPIA web tool. (C) Cell fractionation analysis of AJUBA and TRAF6 cellular distributions in four HCC cells lines. (D) S-tag-AKT, Flag-TRAF6 and HA-ubiquitin were transfected to SNU-449-vector and SNU-449-AJUBA stable cells. Cells were treated with 10 μM MG132 for 8 h before harvest. Cell lysates were immunoprecipitated with S-tag antibody and immunoblotted as indicated. (E) S-tag-AKT, Flag-TRAF6 and HA-ubiquitin were transfected to SNU-449-shNC and SNU-449-shAJUBA stable cells. Cells were treated with 10 μM MG132 for 8 h before harvest. Cell lysates were immunoprecipitated with S-tag antibody and immunoblotted as indicated. TRAF6 knockdown and overexpression efficiencies were determined by qRT-PCR (F, H) and western blotting (G, I) in SNU-449 cells. Three independent experiments were performed. *$P < 0.05$, **$P < 0.01$. Abbreviations: siNC, negative control small interfering RNA; siTRAF6, TRAF6 small interfering RNA.

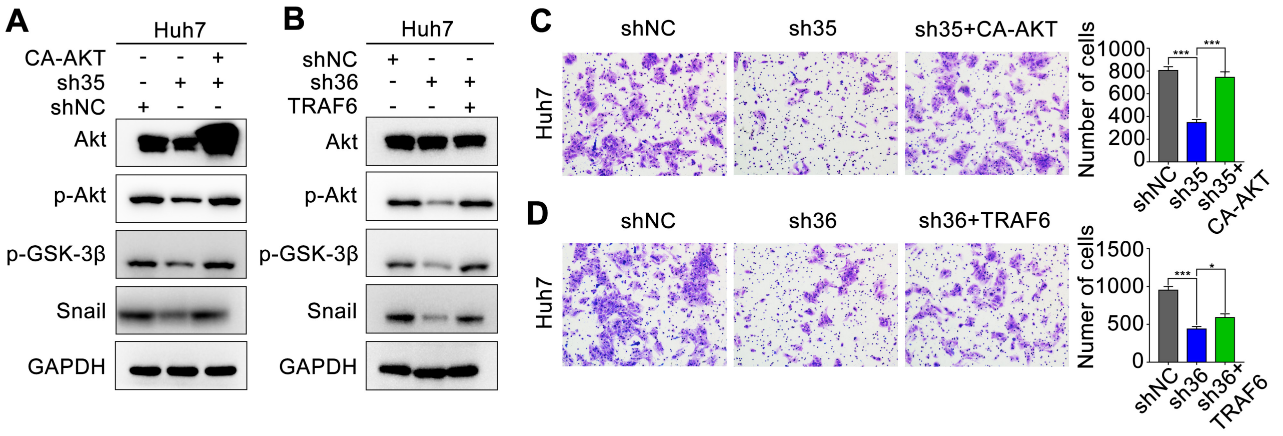

Figure S7. TRAF6 is involved in AJUBA-activated Akt/GSK-3β/Snail pathway in Huh7 cells. (A) Western blotting demonstrated that CA-AKT transfection effectively increased p-Akt, p-GSK-3β, and Snail expression after AJUBA silencing. (B) p-Akt, p-GSK-3β, and Snail expression, which were decreased AJUBA knockdown, were effectively rescued by TRAF6 upregulation. (C) Transwell assay showed that CA-AKT could effectively promote cell migration ability after AJUBA silencing. The right panel shows bar charts of cell migration quantification. Three independent experiments were performed. All error bars show standard error of the mean. ****P* < 0.001. (D) Transwell assay showed that silencing AJUBA inhibited cell migration, which could be reversed by TRAF6 upregulation. The right panels show bar charts of cell migration quantification. Three independent experiments were performed. All error bars show standard error of the mean. **P* < 0.05, ****P* < 0.001.
